# Corundum Particles as Trypsin Carrier for Efficient Protein Digestion

**DOI:** 10.3390/biotech15010002

**Published:** 2025-12-30

**Authors:** Sarah Döring, Birte S. Wulfes, Aleksandra Atanasova, Carsten Jaeger, Leopold Walzel, Georg Tscheuschner, Sabine Flemig, Kornelia Gawlitza, Ines Feldmann, Zoltán Konthur, Michael G. Weller

**Affiliations:** 1 Federal Institute for Materials Research and Testing (BAM), Richard-Willstätter-Strasse 11, 12489 Berlin, Germany; sarah.doering@bam.de (S.D.); carsten.jaeger@bam.de (C.J.); leopold.walzel@bam.de (L.W.); tscheuschnerg@rki.de (G.T.); sabine.flemig@bam.de (S.F.); kornelia.gawlitza@bam.de (K.G.); ines.feldmann@bam.de (I.F.); zoltan.konthur@bam.de (Z.K.); 2 Biological Toxins, Centre for Biological Threats and Special Pathogens, Robert Koch Institute, Seestraße 10, 13353 Berlin, Germany

**Keywords:** aluminum oxide, enzyme immobilization, recombinant trypsin, protein quantification, antibodies, liquid chromatography, mass spectrometry, trastuzumab

## Abstract

Reusable enzyme carriers are valuable for proteomic workflows, yet many supports are expensive or lack robustness. This study describes the covalent immobilization of recombinant trypsin on micrometer-sized corundum particles and assesses their performance in protein digestion and antibody analysis. The corundum surface was cleaned with potassium hydroxide, silanized with 3-aminopropyltriethoxysilane and activated with glutaraldehyde. Recombinant trypsin was then attached, and the resulting imines were reduced with sodium cyanoborohydride. Aromatic amino acid analysis (AAAA) estimated an enzyme loading of approximately 1 µg/mg. Non-specific adsorption of human plasma proteins was suppressed by blocking residual aldehydes with a Tris-glycine-lysine buffer. Compared with free trypsin, immobilization shifted the temperature optimum from 50 to 60 °C and greatly improved stability in 1 M guanidinium hydrochloride. Activity remained above 80% across several reuse cycles, and storage at 4 °C preserved functionality for weeks. When applied to digesting the NISTmAb, immobilized trypsin provided peptide yields and sequence coverage comparable to soluble enzyme and outperformed it at elevated temperatures. MALDI-TOF MS analysis of Herceptin digests yielded fingerprint spectra that correctly identified the antibody and achieved >60% sequence coverage. The combination of low cost, robustness and analytical performance makes corundum-immobilized trypsin an attractive option for research and routine proteomic workflows.

## 1. Introduction

Immobilization of enzymes is a proven strategy to improve their technical applicability [1,2,3,4]. Key advantages include simplified removal after the enzymatic step, enhanced thermal and chemical stability, and the possibility of reuse. These features are particularly relevant where contamination of the product with enzyme or degradation products must be avoided, or where enzyme costs are a limiting factor [5,6,7]. Accordingly, immobilized enzymes have found widespread use both in industrial processes [4,8,9,10] and in analytical applications, such as biosensors [11,12] or proteomics [13,14,15], where enzyme purity and stability are crucial.

Various solid enzyme supports have been explored over the last years in the industry, including acrylate/methacrylate resins [16], agarose [17], (DEAE-) cellulose [18], silica [19,20], and alginates [4,21]. More recently, highly pure corundum (α-Al_2_O_3_, aluminum oxide) has been identified as a promising alternative carrier material [22,23] in biotechnology, for example in affinity purification of proteins. Its advantages include extremely low cost, straightforward surface modification, physical and chemical robustness, fast mass transfer kinetics, negligible leaching of impurities, quick separation due to high density, regenerability, and low non-specific adsorption. In addition, the availability of different grain sizes enables applications ranging from suspension digests to packed-bed enzyme reactors.

To enable stable covalent coupling, surface functionalization of the carrier is essential. A well-known strategy relies on silanization with 3-aminopropyltriethoxysilane (APTES), which introduces primary amine groups [24,25]. These can be further linked via bifunctional aldehyde crosslinkers such as glutaraldehyde [26,27]. The resulting imine bonds are commonly stabilized by gentle reduction with sodium cyanoborohydride (NaCNBH_4_), which converts them into more stable secondary amine linkages [28,29]. This well-established chemistry ensures high coupling efficiency and robust attachment of enzymes.

Trypsin was selected as model enzyme because of its central role in proteomics, where it is considered the “gold standard” for protein digestion [30,31,32,33]. Proteotypic peptides are identified or quantified using liquid chromatography (LC) and/or mass spectrometry (MS) [34,35]. In peptide-based quantification, proteins are enzymatically digested into defined surrogate peptides that uniquely represent the target protein. Quantification is achieved by comparing the LC–MS/MS signal intensities of these peptides with those of spiked isotope–labeled analogs in known amounts, allowing precise determination of the protein amount [35,36,37]. In the context of protein identification, enzymatically generated peptides can be analyzed by peptide mass fingerprinting (PMF), where experimental peptide masses are compared with theoretical in silico digests to enable rapid and reliable protein assignment [31,38,39]. Recombinant production of trypsin further provides key advantages, including the absence of contaminating chymotryptic activity, improved purity, and the possibility to introduce stabilizing modifications [35,40,41,42]. Immobilization of trypsin has been investigated in various contexts before, including agarose or silica beads [17,43], magnetic particles [44,45,46], chitosan supports [47,48], and immobilized enzyme reactors (IMERs) [13,49,50,51,52,53]. Some of these studies have shown that surface attachment can enhance enzyme stability, particularly under elevated temperatures. Compared to commercial silica or magnetic supports that can be expensive and prone to internal diffusion limitations, corundum provides a cost-efficient and non-porous alternative support with tunable particle size and minimal diffusion barriers [22,23].

Based on this knowledge, corundum offers a particularly attractive platform for covalent enzyme immobilization and making it an attractive choice for robust and cheap protein digestion systems. The small particles (~2 µm) exhibit excellent dispersion and homogenization, making them particularly advantageous for suspension-based workflows, e.g., protein digestion, and might be adapted for use in enzyme reactors. In addition to our previous work [22,23], the present study demonstrates the first application of corundum particles as a covalent carrier for an enzyme. After systematic characterization of surface modification and enzyme loading, the particles were assessed with respect to enzymatic activity, thermal and chemical stability, and reusability. To demonstrate practical applicability, immobilized trypsin was applied to two complementary analytical workflows: peptide-based quantification of therapeutic antibodies via LC–MS/MS and antibody identification by MALDI-TOF MS fingerprinting [54,55]. These applications highlight the potential of corundum-based immobilization to combine robustness with analytical benefit in proteomics.

## 2. Materials and Methods

### 2.1. Materials

Calcium chloride (CaCl_2_, 2461.0500), dimethylsulfoxide (DMSO, 2380), ethanol (≥99.9%, 2246.2500), formaldehyde (37%, 2137), hydrochloric acid super pure (34–37%, 7647-01-0), imidazole (9128.0500), LB medium (Miller, 8822), silver nitrate (99%, 7761-88-8), sodium chloride (1367-5KG), toluene (572), tris(hydroxymethyl)aminomethane (8085), tryptone (8028), yeast extract (9263) were purchased from Th. Geyer GmbH & Co. KG (Renningen, Germany). 3-Aminopropyltriethoxysilan (≥98%, 2328.2, APTES), L-arginine (3144.3), cysteine (1693.2), glycerol (4043.1), guanidinium hydrochloride (0037.3), L-lysine monohydrate (A4207), sodium dodecyl sulfate (99.5%, 8029.3), and sodium thiosulfate (≥99%, water free, HN25.1) were obtained from Carl Roth GmbH & Co. KG (Karlsruhe, Germany). Amino acid standard (Supelco, AAS18), cystine (C8755-100G), ethylenediaminetetraacetic acid (EDTA) disodium salt dihydrate (EDS-100G), glutaraldehyde 50% in water (111-30-8), glycine (99%, G7403), di-sodium hydrogen phosphate dihydrate (71643), sodium dihydrogen phosphate anhydrous (71505), ninhydrin (33437-10G), Nα-benzoyl-L-arginine-4-nitroanilide hydrochloride (B3279-500MG), phenol (≥99.5%, P5566-100G), potassium dihydrogen phosphate anhydrous (60218), tris(hydroxymethyl) aminomethane hydrochloride (T5941), and Tween 20 (P1379) were purchased from Sigma-Aldrich (St. Louis, MO, USA). PBS (10× Dulbecco’s powder, A0965.1000) and iodoacetamide (A1666.0025) were obtained from Applichem GmbH (Darmstadt, Germany). Chemicals from Merck KGaA (Darmstadt, Germany) included bromophenol blue (76-59-5) and glucose monohydrate (1.08342.2500). Formic acid (45640.01), kanamycin (26899.03), Quick Coomassie Stain (35081.01), and sodium carbonate (30181.02) were purchased from Serva Electrophoresis GmbH (Heidelberg, Germany). Potassium cyanide (≥98%, 60178-25G) and potassium hydroxide solution (45% in water, 03564-500 ML) were obtained from Honeywell Fluka (Seelze, Germany). Pierce™ C18 tips (10 µL, 87784), sodium cyanoborohydride 95% (25895-60-7), Spectra™ Multicolor Broad Range Protein Ladder (26634), and trifluoroacetic acid (99.5%, 85183) were purchased from Thermo Fisher Scientific (Waltham, MA, USA). Urea (199.075.600) was obtained from AnalytiChem GmbH (Duisburg, Germany). Tris(2-carboxyethyl)phosphine hydrochloride 98% (TCEP, 580560) was purchased from Calbiochem (San Diego, CA, USA). 2,5-Dihydroxyacetophenone (DHAP, A12185) was obtained from Alfa Aesar (Haverhill, MA, USA). ESI (+) calibration standard 5600 (4463272) was purchased from AB Sciex Instruments (Marlborough, MA, USA). LC-MS grade acetonitrile was obtained from Biosolve (Valkenswaard, The Netherlands). Schiff’s reagent (11686.00250) was purchased from Morphisto GmbH (Offenbach, Germany), and human plasma was obtained from DRK-Blutspendedienst West (Münster, Germany).

The NIST monoclonal antibody reference material 8671 (NISTmAb 8671, Batch 14HB-D-002, Sigma Aldrich, St. Louis, MO, USA) [56] and therapeutic, recombinant antibody Herceptin (Trastuzumab, Batch H5207H04, Evidentic GmbH, Potsdam, Germany) were used for protein digestion experiments. The isotopically labeled peptide standard (ALPAPIEK-^13^C,^15^N, mass shift + 8 amu) was synthesized by PSL Peptide Specialty Laboratories GmbH (Heidelberg, Germany) and purity was confirmed by HPLC-UV. Corundum F1500 was obtained by Haixu Abrasives (Zhengzhou, China). Ultrapure water was used from a Milli-Q water purification system (Millipore, Bedford, MA, USA) with a resistivity of >18.2 Ω and TOC value of <5 ppm.

### 2.2. Particle Characterization via TEM, ESEM, DLS and Particle Size Distribution

Transmission electron microscopy (TEM) images and energy-dispersive X-ray spectroscopy (EDS) analyses were obtained using a Talos F200S instrument operated at 200 kV (Thermo Fisher Scientific, Waltham, MA, USA). For sample preparation, 10 µL of a 1 mg/mL corundum suspension in ethanol was deposited onto a carbon-coated copper grid and air-dried at room temperature (RT).

Environmental scanning electron microscopy (ESEM) was conducted using an XL 30 ESEM equipped with a tungsten cathode (FEI, Eindhoven, The Netherlands) and electronically upgraded by Point Electronic GmbH (Halle, Germany). For ESEM analysis, 15 µL of a 1 mg/mL corundum suspension in ethanol was placed onto aluminum SEM stubs and subsequently sputter-coated with a 15 nm gold layer. Imaging was carried out in high-vacuum mode using a secondary electron detector.

Dynamic light scattering (DLS) experiments were performed at an Anton Paar Lite-Sizer 500 instrument with disposable cuvettes made from polystyrene (10 mm) purchased from Th. Geyer GmbH & Co. KG (Renningen, Germany). Measurements were performed with 1 mg/mL corundum dispersed in ultrapure water, using forward scatter mode and the advanced cumulant model.

Particle size distribution of corundum F1500 was measured by the manufacturer, Haixu Abrasives (Zhengzhou, China), using a laser-based particle size analyzer LS-POP (6) from Zhuhai OMEC Instruments Co., Ltd. (Zhuhai, China).

### 2.3. Recombinant Production of Porcine Trypsin

As a representative proteolytic enzyme, recombinantly produced porcine trypsin was used for immobilization onto APTES- and glutaraldehyde-functionalized corundum particles. The coding sequence was derived from the native porcine trypsinogen gene (UniProt ID: P00761). A detailed protocol describing the expression in *E. coli*, protein refolding, enzymatic activation, and purification steps are provided in the Supplementary Material.

### 2.4. Surface Modification and Trypsin-Immobilization of Corundum

All centrifugation steps were performed at 10,000× *g* for 5 min at RT and incubation of corundum suspension was always performed in an overhead rotator at 30 rpm. An amount of 1 g raw corundum particles was incubated in 5 mL of 10% (*v*/*v*) potassium hydroxide solution for a brief cleaning of the surface at RT for 10 min. After centrifugation, the supernatant was discarded, the corundum was washed with ultrapure water twice and dried under vacuum overnight. For silanization, the dried corundum particles were washed twice with toluene, then incubated with 1% APTES (*v*/*v*) solution in 99:1 toluene–water for 16 h at RT. On the following day, the corundum was washed three times with toluene and dried under vacuum at 120 °C for 3 h. The silanized corundum was then flushed with argon and stored at 4 °C until further use.

For enzyme immobilization, 100 mg of the silanized corundum particles were incubated with 2 mL of 5% (*v*/*v*) glutaraldehyde solution in ultrapure water for 1 h at RT, followed by three washing steps with ultrapure water and two washing steps with PBS (pH 7.4). Coupling of in-house produced trypsin (1 mL, 2 mg/mL) was performed over 16 h at 4 °C. Without intermediate washing, the particles were reduced with 2 mL of a 50 mM NaCNBH_4_ solution and kept in an overhead shaker for 1 h at RT. After three washing steps with PBST (0.1% Tween 20, pH 7.4) and one wash with PBS, the trypsin particles were blocked by incubation in 1 mL of TGL-blocking buffer (1 M Tris, 0.5 M glycine, 0.5 M lysine, pH 7.4) for 1 h at RT. Finally, the particles were reduced again with NaCNBH_4_, washed three times with PBST (0.1% Tween 20) and once with PBS. The functionalized particles were then ready for further experiments.

### 2.5. Colorimetric Verification of Surface Functionalization

#### 2.5.1. Kaiser Test

The presence of free primary amine groups on the functionalized corundum surface was assessed using the Kaiser test [57,58,59]. For each sample, 10 mg of dry corundum was mixed with 200 µL of solutions A (50 mg/mL ninhydrin in ethanol), B (800 mg/mL phenol in ethanol) and C (1:50 diluted 0.065 mg/mL potassium cyanide solution in ultrapure water). The suspension was incubated for 5 min at 90 °C and 1000 rpm in an Eppendorf ThermoMixer C. After centrifugation at 10,000× *g* for 5 min, 200 µL of the supernatant was transferred to a cuvette and the absorbance of the resulting Ruhemann’s purple was measured photometrically at 586 nm. A reagent blank containing only solutions A, B, and C was used as a control.

#### 2.5.2. Schiff Test

To verify successful surface functionalization with glutaraldehyde, the Schiff test was applied. For this purpose, 10 mg of dry corundum was mixed with 400 µL of Schiff’s reagent and incubated for 5 min at RT in a ThermoMixer C at 1000 rpm. After centrifugation at 10,000× *g* for 5 min, the supernatant was removed, and the particles were washed twice with ultrapure water prior to visual inspection.

### 2.6. Aromatic Amino Acid Analysis (AAAA)

Aromatic amino acid analysis (AAAA) was performed following a previously described protocols with minor modifications [22,60]. A volume of 100 µL of the trypsin-functionalized corundum suspension, corresponding to 100 mg of corundum particles, was transferred to hydrolysis-grade glass tubes and mixed with 900 µL of 37% hydrochloric acid. The tubes were purged with an argon stream for 60 s to displace atmospheric oxygen. Hydrolysis was carried out at 150 °C for 1 h in a copper heating block under continuous magnetic stirring. After cooling down to RT and centrifugation at 10,000× *g* for 5 min, 200 µL of the supernatant was transferred to a 1.5 mL reaction tube and evaporated at 40 °C and 1 mbar using a vacuum concentrator (Martin Christ, Osterode, Germany) for 16 h. The dried residue was reconstituted in 200 µL of ultrapure water, centrifuged at 10,000× *g* for 15 min, and the supernatant was transferred to HPLC vials for analysis.

Chromatographic separation was performed on a 1260 Infinity II HPLC system (Agilent Technologies, Santa Clara, CA, USA) operated with OpenLAB CDS software (version A.04.06). For analysis, an AdvanceBio AAA column (3.0 × 100 mm, 2.7 µm particle size) equipped with a corresponding guard column (3 × 5 mm, 2.7 µm) was used at 40 °C. Tyrosine was detected fluorometrically using a fluorescence detector (FLD) at an excitation wavelength of 272 nm and emission at 303 nm. Isocratic separation was performed over 10 min using a mobile phase consisting of 99% eluent A (Ultrapure water, 0.1% TFA) and 1% eluent B (ACN, 0.1% TFA). This was followed by a column washing step using 90% eluent B and 10% eluent A for 10 min. Column equilibration was performed under the initial separation conditions. Quantification was based on external calibration using an amino acid standard mixture.

### 2.7. Sodium Dodecyl Sulfate-Polyacrylamide Gel Electrophoresis (SDS-PAGE)

To evaluate non-specific protein adsorption onto functionalized particles, SDS-PAGE was performed using an XCell SureLock electrophoresis system (Invitrogen AG, Waltham, MA, USA) with a Novex WedgeWell 8–16% Mini Protein Gel (Tris-glycine, 1.0 mm, 15-well), as previously described [22]. All centrifugation steps were carried out at 10,000× *g* for 5 min at RT and the corundum suspension was always incubated in an overhead rotator at 30 rpm. An amount of 100 mg raw and functionalized corundum particles was incubated with 1 mL of 1:10 diluted, sterile-filtered (0.22 µm) human plasma in PBS for 1 h at 4 °C. Following incubation, the particles were washed three times with PBST (0.1% Tween 20) and twice with PBS. To elute non-specifically bound proteins, the corundum samples were incubated with 125 µL SDS loading buffer (4× concentrated: tris base (252 mM, pH 6.8), glycerol (40%), SDS (8%), and bromophenol blue (0.02%), in ultrapure water) and 375 µL PBS for 15 min at RT, after which the supernatant was collected. As reference samples, 18 µL of either 1:10 diluted human plasma or trypsin were mixed with 6 µL of 4× SDS loading buffer. All samples were heated up to 90 °C for 5 min and centrifuged briefly before loaded onto the gel. Electrophoretic separation of proteins was performed at 70 V for 10 min and 180 V for another 60 min. The protein staining was performed using Quick Coomassie Stain (Serva Electrophoresis GmbH, Heidelberg, Germany) for 30 min and subsequent washing with ultrapure water over night.

### 2.8. Trypsin Activity Assay Using Benzoyl-DL-arginine-p-nitroanilide (BAPNA)

The enzymatic activity of immobilized trypsin was determined by monitoring the hydrolysis of Nα-benzoyl-DL-arginine-p-nitroanilide (BAPNA) [61] to p-nitroaniline at 405 nm using a NanoPhotometer NP80 (Implen GmbH, Munich, Germany). A 50 mM BAPNA stock solution was prepared in dimethyl sulfoxide (DMSO) and subsequently diluted with 25 mM Tris buffer (pH 8.0) to obtain a final concentration of 500 µM.

For standard activity assays, 500 µL of the BAPNA solution was added to 10 mg of trypsin-functionalized corundum particles (corresponding to 9.8 µg immobilized trypsin) and immediately vortexed. The suspensions were incubated at room temperature on an overhead rotator at 30 rpm for defined time points (5, 10, 15, 20, 30, and 60 min). After each incubation, samples were centrifuged at 10,000× *g* for 2 min, and 100 µL of the supernatant was transferred to a cuvette for absorbance measurement. The formation of p-nitroaniline was calculated based on the absorbance at 405 nm using the Lambert–Beer law (ε = 9960 L mol^−1^ cm^−1^, path length = 1 cm). A substrate solution without enzyme served as blank control. After each measurement, the 100 µL aliquot was returned to the reaction tube, vortexed for 1 min, and the incubation continued. One unit (U) of trypsin activity was defined as the amount of enzyme catalyzing the conversion of 1 µmol of BAPNA substrate per minute at RT with 25 mM Tris buffer (pH 8.0).

To assess temperature-dependent activity, particle suspensions were incubated for 15 min at various temperatures (37 °C, 50 °C, 60 °C, 70 °C, and 80 °C), followed by centrifugation and absorbance measurement as described above. For denaturation stability testing, trypsin-functionalized particles were incubated overnight in 1 M guanidinium hydrochloride (Gdn-HCl) at RT, 37 °C and 50 °C. After washing, residual activity was determined by incubation of particles with 500 µM BAPNA solution for 15 min. All activity and stability experiments were also conducted using 2.0 µg of free (non-immobilized) trypsin in solution under identical conditions as a reference.

Reusability was evaluated by performing repeated incubations with 500 µL of the BAPNA solution for 15 min at 37 °C and 50 °C using the same particle batch. After each cycle, the particles were washed thoroughly twice with PBS before being reused in the next reaction.

### 2.9. Antibody Digestion and LC-MS/MS-Based Quantitative NISTmAb Analysis

Unless otherwise stated, all reagents were prepared in 0.1 M Tris buffer (pH 7.8), consisting of 0.394 g Tris(hydroxymethyl)aminomethane and 1.064 g Tris(hydroxymethyl) aminomethane hydrochloride dissolved in 100 mL ultrapure water. All reactions were carried out in 1.5 mL low-protein-binding Eppendorf tubes.

For denaturation and reduction of the antibody, 5 µL (50 µg) of NIST monoclonal antibody reference material 8671 (NISTmAb 8671, 10 mg/mL) [56] were mixed with 42 µL of 8 M Gdn-HCl and 140 mM Tris(2-carboxyethyl)phosphine (TCEP), resulting in final concentrations of 6 M Gdn-HCl and 5 mM TCEP. Additionally, 2 µL of a ^13^C,^15^N-isotopically labeled ALPAPIEK peptide standard (0.081 mg/mL) was added. The mixture was incubated at 37 °C for 30 min in an Eppendorf ThermoMixer C at 1000 rpm.

For alkylation of cysteines, 4 µL of 180 mM iodoacetamide in 0.1 M Tris buffer (pH 7.8) was added to reach a final concentration of 12 mM in a total volume of 60 µL. The reaction was incubated in the dark at RT for 30 min at 1000 rpm.

For antibody digestion using immobilized trypsin, 10 mg of trypsin-functionalized corundum particles were suspended in 300 µL of 0.1 M Tris buffer (pH 7.8) and added to the sample to achieve an antibody-to-trypsin ratio of 1:5 (*w*/*w*), ensuring a final Gdn-HCl concentration below 1 M. For comparison, digestion with free trypsin in solution was performed by adding 290 µL of 0.1 M Tris buffer (pH 7.8) and 10 µL of trypsin stock solution (0.25 mg/mL) to the reduced and alkylated antibody solution, resulting in a final enzyme-to-substrate ratio of 1:20 and <1 M Gdn-HCl. All digestions were carried out in an Eppendorf ThermoMixer C at different temperatures (37 °C, 50 °C, 60 °C and 70 °C) at 1400 rpm. At predefined time points, 90 µL aliquots were withdrawn, mixed with 10 µL of 2% formic acid (FA, final concentration 0.1%) to stop the enzymatic digestion, and centrifuged at 10,000× *g* for 20 min at 4 °C.

For reusability experiments, the digestion occurs for full 16 h at 37 °C and the supernatant volume of 270 µL were mixed with 30 µL of 2% FA (final concentration 0.1%) to stop the enzymatic digestion. The particles were washed three times with PBST and twice with 0.1 M Tris buffer (pH 7.8) before reuse. All supernatants were stored at –20 °C until LC-MS/MS analysis.

Tryptic digests were analyzed using a 1290 Infinity II UHPLC system (Agilent Technologies, Waldbronn, Germany) coupled to a TripleTOF 6600 mass spectrometer (AB Sciex Instruments, Marlborough, MA, USA). For each run, 5 µL of the digested antibody sample (corresponding to 0.625 µg total protein) were injected in randomized order onto an ACQUITY Premier BEH C18 column (2.1 × 100 mm, 1.7 µm particle size; Waters, Milford, MA, USA), maintained at 50 °C. Chromatographic separation was performed using gradient elution over 13 min, starting with 99% eluent A (Ultrapure water, 0.1% FA) and 1% eluent B (ACN, 0.1% FA), and ending with 50% eluent A and 50% eluent B. The column was flushed with 99% eluent B for 2 min, followed by a 3 min re-equilibration at starting conditions. The flow rate was set to 0.6 mL/min. Samples were measured in biological duplicates.

Electrospray ionization (ESI) was performed in positive (+) ion mode with a capillary voltage of +5500 V and a source temperature of 450 °C. MS spectra were acquired over an *m/z* range of 100–1200. MS/MS data were collected in data-independent acquisition (DIA) mode using a sequential windowed acquisition of all theoretical fragment ion mass spectra (SWATH) technique with 11 predefined variable isolation windows (see Supplementary Table S1). The total cycle time was 0.8 s. Mass calibration was conducted at the beginning of the sequence using an ESI (+) calibration standard 5600 (AB Sciex Instruments, Marlborough, MA, USA) and repeated after every tenth sample.

For data processing, raw MS files were imported into MS-DIAL (Version 4.9.221218) [62]. The analysis was performed using the following parameters: soft ionization, data-independent MS/MS (SWATH), profile data, positive ion mode, and metabolomics workflow settings. Detailed analysis settings were left at default, except for retention time end (10 min), surrogate and isotopic labeled peptide identification retention time tolerance (1 min) and identification score cut off (60%) with NIST mAb spectral library [63].

### 2.10. MALDI-TOF MS-Based Antibody Fingerprinting and Identification of Herceptin

The enzymatic digestion and MALDI-TOF MS analysis were performed following a previously published protocol for antibody fingerprinting with minor modifications [55]. In brief, 25 µg of Herceptin (Batch H5207H04, Evidentic GmbH, Potsdam, Germany) was mixed with 0.1 mM TCEP in 0.1 M Tris buffer (pH 7.8) to a final volume of 75 µL in low-protein-binding 1.5 mL Eppendorf tubes. Antibody denaturation and disulfide bond reduction were conducted at 99 °C for 15 min at 950 rpm in an Eppendorf ThermoMixer C. No alkylation step was included. Following thermal treatment, the samples were cooled to 37 °C or 60 °C. For enzymatic digestion, free trypsin was added to a final volume of 125 µL to achieve a trypsin-to-antibody mass ratio of 1:120. In comparison, 1 mg of trypsin-functionalized corundum particles was suspended in 50 µL of 0.1 M Tris buffer (pH 7.8) and added to reach an antibody-to-trypsin ratio of 1:30 (*w*/*w*). Digestions were carried out for 60 min at 37 °C and 1400 rpm. Subsequently, 25 µL of 0.1% TFA was added to stop the reaction. Peptides were enriched and desalted using Pierce™ C18 tips (10 µL) according to the manufacturer’s protocol. Elution was performed directly onto a MALDI target plate using 2 µL of 2,5-dihydroxyacetophenone (DHAP) matrix solution (10 mg/mL in 69.9% ultrapure water, 30% acetonitrile, 0.1% TFA). MALDI-TOF MS spectra were acquired using a Autoflex maX (Bruker Daltonics GmbH & Co. KG, Bremen, Germany) instrument operated in reflector mode. External calibration was performed using the Bruker Peptide Calibration Standard II with DHAP as the matrix. A total of 5000 laser shots were accumulated per sample to generate fingerprint spectra.

Peptide mass fingerprints were analyzed using the web-based software ABID 2.0 (developed by Jan Lisec, BAM, Berlin [64]; https://bam.de/ABID, last accessed on 6 October 2025) to compare the particle-digested antibody Herceptin with a positive control (digest with free trypsin) and to identify matches from a reference library of over 70 antibody spectra. Matching is based on the number of overlapping peptides between the sample spectrum and library entries. In addition to peptide overlaps, ABID also calculates an intensity-weighted score, in which matches of high-intensity peaks are weighted more strongly than those of lower intensity. The parameter “dmz” was set to 0.3 Da and 10 ppm, with a signal-to-noise ratio (SNR) threshold of 12. ABID 2.0 allows online analysis without installation and is described in detail elsewhere [55]. The source code is available at https://github.com/BAMresearch/ABID (last accessed on 6 October 2025).

## 3. Results

### 3.1. Characterization of Raw and Surface-Modified Corundum Particles

In this study, the unmodified corundum particles were first examined using TEM and ESEM to verify structural integrity and particle homogeneity prior to functionalization. Based on initial handling tests, the F1500 particle fraction was selected because it formed stable pellets during centrifugation and allowed particle-free removal of supernatant. This fraction also exhibited reduced sedimentation during mixing and could be easily dispersed, ensuring a homogeneous and reproducible workflow. In contrast to previously described materials that exhibited production-related needle-like structures and surface irregularities [22], the corundum used here shows high purity and morphological uniformity.

**Figure 1:**
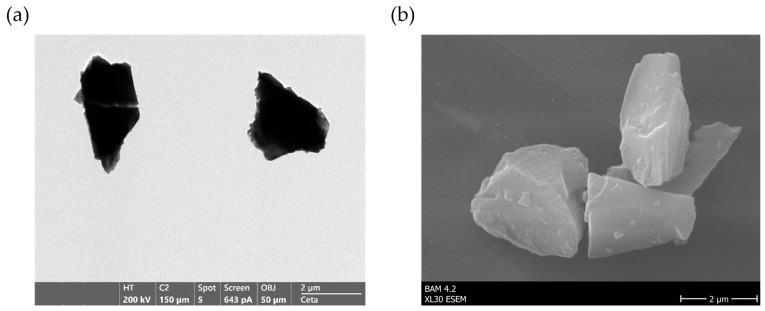
Morphological characterization of raw corundum particles. (**a**) TEM images show angular shapes without obvious impurities. (**b**) ESEM micrograph illustrating the nonporous material.

TEM images showed the expected angular shape with smooth particle surfaces (Figure 1a). ESEM micrographs confirmed a homogeneous particle population free from visible contaminants (Figure 1b). The particles appeared non-porous, with sharply defined edges and compact geometry, consistent with the dense structure of fused aluminum oxide. Visual inspection of the TEM images (Figure 1b) revealed that the particles had an average diameter of approximately 2 µm. This observation agrees well with the manufacturer’s particle size data (D10 = 1.54 µm, D50 = 2.21 µm, D90 = 2.97 µm; Figure S1) and dynamic light scattering (DLS) measurements (Figure S2), which indicated an average particle size of around 1.55 ± 0.27 µm. These results confirm the relatively narrow and homogeneous size distribution of the corundum material. To further assess the elemental composition, energy-dispersive X-ray spectroscopy (EDS) was performed (Figure S3). The detected signals were aluminum (42.9%), oxygen (48.2%) and minor carbon and copper signals were attributed to the carbon support film and the TEM grid, respectively. No signals indicative of foreign elements or surface-bound salts were detected.

**Figure 2:**
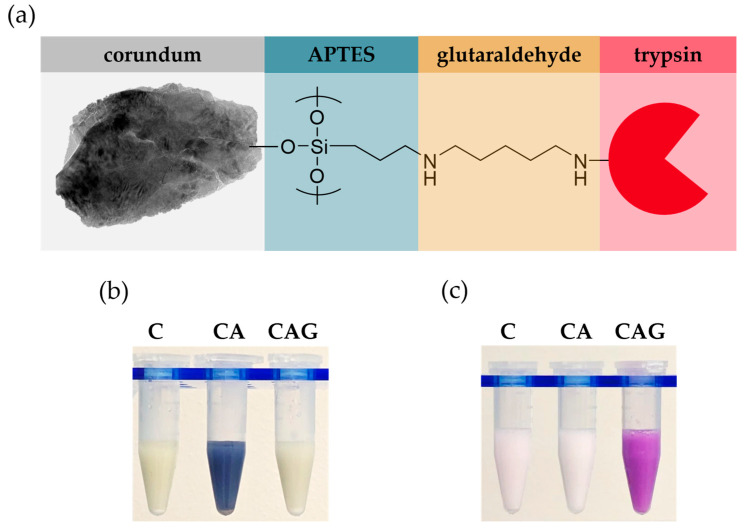
Immobilization scheme and verification of surface functional groups on raw corundum (C), APTES-silanized particles (CA), and glutaraldehyde-modified particles (CAG). (**a**) Schematic illustration of the functionalization strategy including APTES silanization, glutaraldehyde crosslinker (reduced), and enzyme conjugation via lysine. (**b**) Kaiser test (ninhydrin-based) for the detection of primary amine groups. (**c**) Schiff test for the detection of surface-bound aldehyde groups.

For covalent enzyme coupling, the corundum surface was first functionalized with the amino silane APTES, followed by modification with the formally bifunctional crosslinker glutaraldehyde and subsequent enzyme immobilization (Figure 2a). In contrast to previously described silanization protocols that employed ethanol as solvent [22,23], toluene was used here due to its lower cost and comparable performance. Successful APTES functionalization was confirmed using the Kaiser test, in which the reaction of ninhydrin with terminal amine groups produces a deep violet color (Ruhemann’s purple) in the supernatant [57,58,59]. No color change was observed for untreated corundum, whereas APTES-functionalized particles exhibited a pronounced colourimetric response (Figure 2b). Photometric quantification at 586 nm indicated that only 2% of amine groups remained detectable after glutaraldehyde treatment (Table S2). Raw corundum produced some background noise, based on device-specific accuracy.

To verify glutaraldehyde modification, the Schiff test was applied. This assay is based on the reaction of aldehyde groups with decolorized Schiff’s reagent, restoring its chromophor and forming a pink Schiff base [65,66]. In the present case, the coloration developed directly on the particle surface, indicating the presence of surface-bound aldehyde groups (Figure 2c). In contrast, raw or APTES-functionalized particles showed no visible staining.

**Figure 3:**
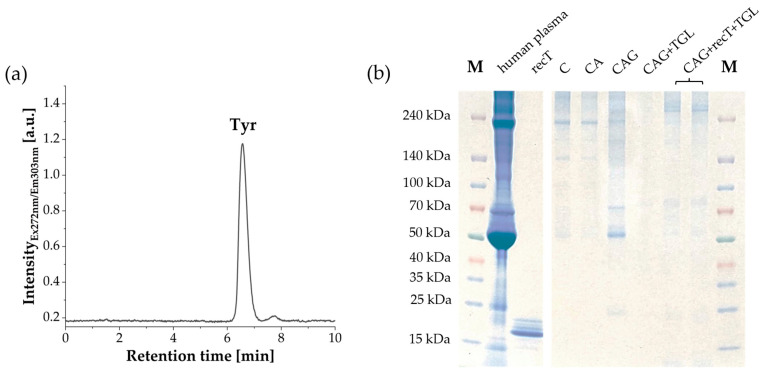
Characterization of enzyme loading by aromatic amino acid analysis (AAAA) and non-specific protein binding of human plasma on corundum with different surface modifications. (**a**) HPLC chromatogram of released tyrosine detected by fluorescence (excitation 272 nm, emission 303 nm), calibration see Supplementary Material Figure S4. (**b**) SDS-PAGE analysis of non-specific binding after incubating raw corundum (C), APTES-functionalized (CA), glutaraldehyde-modified (CAG), TGL-blocked (CAG + TGL), and trypsin-immobilized (CAG + recT + TGL) particles with human plasma, followed by washing and elution with 2% SDS.

In the next step, the enzyme trypsin was covalently immobilized onto the corundum surface via glutaraldehyde crosslinking, which reacts non-selectively with ε-amino groups of lysine residues accessible on the enzyme surface. The successful immobilization of trypsin onto glutaraldehyde-functionalized corundum particles was verified using Aromatic Amino Acid Analysis (AAAA) [67]. This method has previously been shown to be suitable for the direct quantification of protein immobilized on corundum surfaces [22]. The total amount of immobilized trypsin was determined using an external tyrosine calibration curve and the known number of tyrosine residues in the trypsin amino acid sequence (Figure S4). HPLC analysis of the acid hydrolysate showed a distinct tyrosine peak detectable by fluorescence detection (Figure 3a). Based on this approach, the amount of trypsin immobilized on 1 mg of modified corundum was quantified as 0.98 ± 0.02 µg from an initial 20 µg applied. Furthermore, DLS measurements after immobilization indicated a slight increase in particle diameter (Figure S2).

For use in enzymatic protein digestion, non-specific interactions with other proteins should be as low as possible. Therefore, corundum particles at different functionalization stages were incubated with human plasma. After washing with PBST and PBS, adsorbed proteins were eluted with 2% SDS, and the eluates were analyzed by SDS-PAGE (Figure 3b). Coomassie staining showed prominent high-molecular-weight bands in lanes from raw (C) and APTES-silanized corundum particles (CA), indicating non-specific adsorption. After glutaraldehyde modification, band intensity increased further, consistent with covalent attachment of plasma proteins as well as strong non-specific binding [22]. After blocking with a buffer consisting of Tris, glycine and lysine (TGL), no proteins were detected in the SDS eluate by Coomassie staining. When trypsin was immobilized first, and the particles were then blocked, faint bands reappeared in the eluate. Notably, no trypsin band was detected in any eluate after treatment with 2% SDS.

### 3.2. Enzymatic, Temperature-Dependent Activity and Reusability

**Figure 4:**
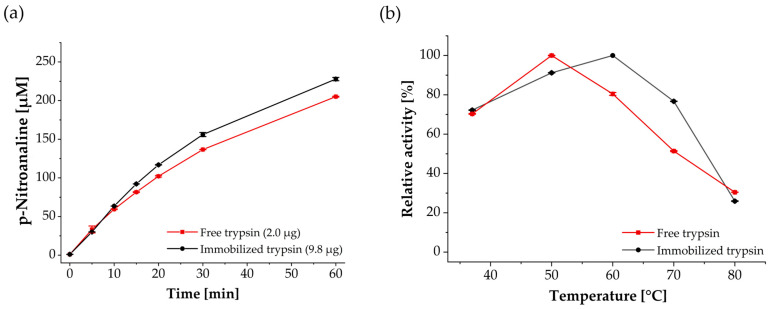
Enzymatic activity of corundum-immobilized trypsin (black) compared to free trypsin (red) using 500 µM BAPNA solution in 25 mM Tris (pH 8.0). (**a**) Immobilized trypsin showed a four-fold lower specific activity (0.31 U/mg trypsin) compared to free trypsin (1.28 U/mg trypsin). (**b**) Relative activity at temperatures ranges from 37 °C to 80 °C. The corundum-immobilized trypsin reached its maximum at 60 °C and retained higher activity at elevated temperatures, whereas the free enzyme peaked at 50 °C. Errors were calculated as the relative standard deviation of duplicate measurements.

After successful immobilization of trypsin onto corundum particles, enzyme activity was evaluated with the Nα-benzoyl-DL-arginine-p-nitroanilide (BAPNA) assay and compared to free trypsin in solution. 10 mg of trypsin-functionalized particles (9.8 µg enzyme) and free trypsin (2.0 µg) were incubated in a substrate solution of 500 µM BAPNA in 25 mM Tris buffer (pH 8.0) at RT over a time period of 60 min. Based on p-nitroaniline formation and photometric absorbance measurements at 405 nm, initial rates and specific activities were calculated in the initial linear range. For corundum-functionalized trypsin, an initial rate of 6.0 µM/min was recorded, corresponding to a specific activity of 0.31 U/mg enzyme (Figure 4a). With free trypsin in solution, the rate was 5.1 µM/min with a specific activity of 1.28 U/mg enzyme. Based on the BAPNA measurements, the immobilized particles showed about 25% of the catalytic activity of free trypsin.

To further evaluate the impact of immobilization on thermal performance, the temperature-dependent activity of free trypsin was compared in solution and immobilized on corundum particles (Figure 4b). When incubated at 37 °C, comparable relative activities of 72.2 ± 0.1% (immobilized trypsin) and 70.3 ± 0.2% (free trypsin) were measured. The maximum activity of corundum-immobilized trypsin was reached at 60 °C, whereas free trypsin showed its optimum already at 50 °C. At 60 °C, the activity of free trypsin declined successively and with increasing temperatures, immobilized trypsin retained more activity than the free enzyme. At 80 °C, only about 30% activity (BAPNA) was detected for both variants.

**Figure 5:**
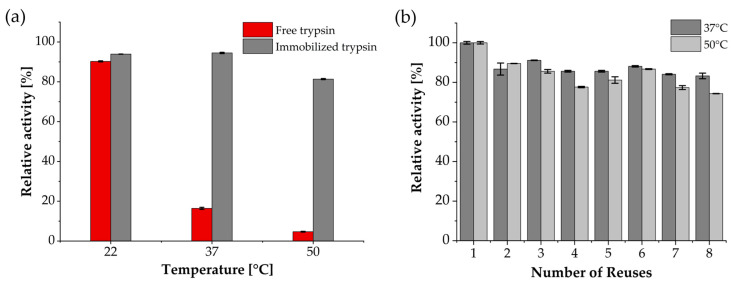
Chaotropic tolerance and reusability of trypsin-functionalized corundum. (**a**) Relative residual activity of free and immobilized trypsin after overnight incubation in 1 M Gdn-HCl at room temperature, 37 °C, and 50 °C. (**b**) Reusability of trypsin-functionalized corundum particles over eight BAPNA assay cycles at 37 °C and 50 °C. Errors were calculated as the relative standard deviation of duplicate measurements.

Particularly in connection with bottom-up protein quantification, enzymatic digestion is often performed under mild denaturing conditions (≤1 M denaturing agent) using urea or guanidinium hydrochloride (Gdn-HCl). To assess stability under such conditions, trypsin-immobilized corundum particles and free trypsin were incubated with 1 M Gdn-HCl in 0.1 M Tris buffer (pH 7.8) for 16 h at RT, 37 °C, and 50 °C. Enzyme activity was measured with 500 µM BAPNA solution at RT for 15 min. Relative activity was calculated based on initial activity prior to incubation with Gdn-HCl and was set to 100%. After incubation overnight in 1 M Gdn-HCl at RT, both free and immobilized trypsin retained high activity with no substantial loss (Figure 5a). In contrast, after incubation at elevated temperatures, free trypsin lost nearly all activity, retaining less than 20% at 37 °C and below 5% at 50 °C. Immobilized trypsin, however, remained highly active, with more than 90% activity at 37 °C and over 80% at 50 °C.

The ability to reuse an enzyme preparation is one of the key advantages of immobilization. This has important economic and practical implications, especially in large-scale or routine protein digestion workflows. To assess this property, trypsin-functionalized corundum particles were repeatedly incubated with 500 µM BAPNA solution at 37 °C and 50 °C. After each cycle, particles were washed with 0.1 M Tris buffer (pH 7.8), resuspended fresh BAPNA solution, and enzyme activity was determined (Figure 5b). At 37 °C, more than 83% of the initial activity was preserved throughout all cycles, whereas at 50 °C activity declined more rapidly and reached only about 74% by the end.

Finally, storage tests showed that trypsin-functionalized corundum particles lost most of their activity at room temperature, whereas storage at 4 °C or lower preserved more than 55% over 4 weeks (Figure S5). This is consistent with stability ranges reported for proof-of-concept applications of other immobilized trypsin systems [44,47].

### 3.3. Application for LC-MS/MS-Based Quantification of NISTmAb

Peptide-based quantification via LC–MS/MS is the standard approach for reliable protein measurement, as it depends on reproducible enzymatic digestion into peptides. To benchmark the performance of corundum-immobilized trypsin, the NISTmAb reference antibody [56] with defined protein content, sequence, and spectral library was chosen as a model system. This setup should demonstrate the suitability of immobilized trypsin for development of accurate proteolytic digestion workflows.

**Figure 6:**
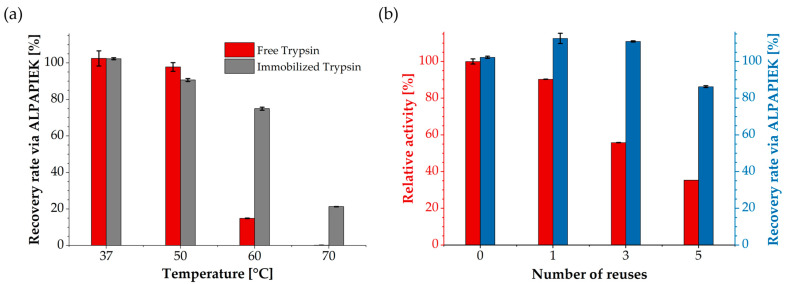
Application of immobilized trypsin for quantitative antibody digestion. (**a**) Recovery rate of NISTmAb via surrogate peptide ALPAPIEK after a 16 h digestion at different temperatures, comparing free and immobilized trypsin. (**b**) Reusability of immobilized trypsin particles over consecutive digests at 37 °C, monitored recovery rate by LC–MS/MS (blue) and residual enzyme activity in the BAPNA assay (red). Errors were calculated as the relative standard deviation of duplicate measurements.

The NISTmAb reference antibody was first denatured and reduced in 6 M Gdn-HCl containing TCEP, followed by alkylation with IAM. After dilution to a final concentration below 1 M Gdn-HCl, the samples were digested using either free trypsin in solution or trypsin immobilized on corundum particles at different incubation temperatures. Quantification was performed via the surrogate peptide ALPAPIEK and its isotope-labeled analog, which is widely used for antibody quantification [68,69,70]. Recovery rate was calculated as the percentage of the measured peptide amount relative to the theoretical yield based on the known antibody input. Under standard conditions at 37 °C, recoveries were nearly identical for free and immobilized trypsin (Figure 6a). While performance was still high at 50 °C, differences emerged at higher temperatures. Free trypsin rapidly lost activity, dropping below 15% recovery rate at 60 °C and becoming almost inactive at 70 °C. In contrast, immobilized trypsin still produced substantial recoveries of about 75% at 60 °C. Additional time-course experiments (Figure S6) further showed that immobilized trypsin was particularly effective at 50 °C in short digests, while extended digestion times resulted in recoveries similar to those at 37 °C. In addition to targeted quantification, sequence coverage analysis confirmed broad proteolytic activity. Comparable values of 71% for immobilized trypsin and 65% for free trypsin were obtained (Tables S3–S6).

To further evaluate their practical applicability, the particles were reused in consecutive antibody digestion cycles at 37 °C overnight. After each run, they were washed with PBST and 0.1 M Tris buffer (pH 7.8) before being reused. The BAPNA assay showed a gradual decrease in activity, from around 90% after the first reuse to about 35% after five cycles (Figure 6b). In contrast, LC–MS/MS-based quantification of NISTmAb showed that the recovery rate of the target peptide ALPAPIEK remained largely stable during the first three cycles and only dropped below 90% after the fifth cycle. Blank runs were performed between selected digestion cycles (runs 2 and 4) to assess potential carryover effects. These control experiments revealed a low ALPAPIEK residual signal of approximately 5–8%, indicating that small amounts of peptide likely remained adsorbed to the particle surface despite washing and were detectable in the subsequent run (Figure S7). Consistent with this interpretation, recovery rates slightly above 100% in antibody-containing runs (runs 1 and 3) can be plausibly explained by a minor contribution from residual peptide together with analytical variability.

### 3.4. Application for MALDI-TOF MS-Based Identification of Herceptin

Beyond quantitative peptide analysis, immobilized trypsin was further evaluated for its ability to generate peptide mass fingerprints suitable for antibody identification using established MALDI-TOF MS methods [55]. The therapeutic antibody Herceptin (Trastuzumab) was selected as a model system due to its therapeutic relevance in cancer treatment [71] and availability of the complete antibody sequence under D03257 at Kyoto Encyclopedia of Genes and Genomes (KEEG, https://www.genome.jp/kegg/, last accessed on 6 October 2025).

Following thermal denaturation and reduction with TCEP in 0.1 M Tris buffer (pH 7.8) at 99 °C, Herceptin was digested either with free trypsin in solution or with trypsin immobilized on corundum particles for one hour. The resulting peptide mixtures were analyzed by MALDI-TOF MS. The fingerprint spectra of free trypsin digest served as positive control, and the resulting spectrum was integrated into a library of over 70 reference spectra. Spectra from immobilized-trypsin digests were matched against this library using the ABID software for automated antibody identification [64]. Besides peptide matches, an intensity-weighted score (column: “intweight”) was applied, giving greater weight to abundant peaks with high intensity. This approach strengthened the peptide assignment of immobilized-trypsin digests to Herceptin reference spectra and minimized the influence of low-intensity mismatches.

**Figure 7:**
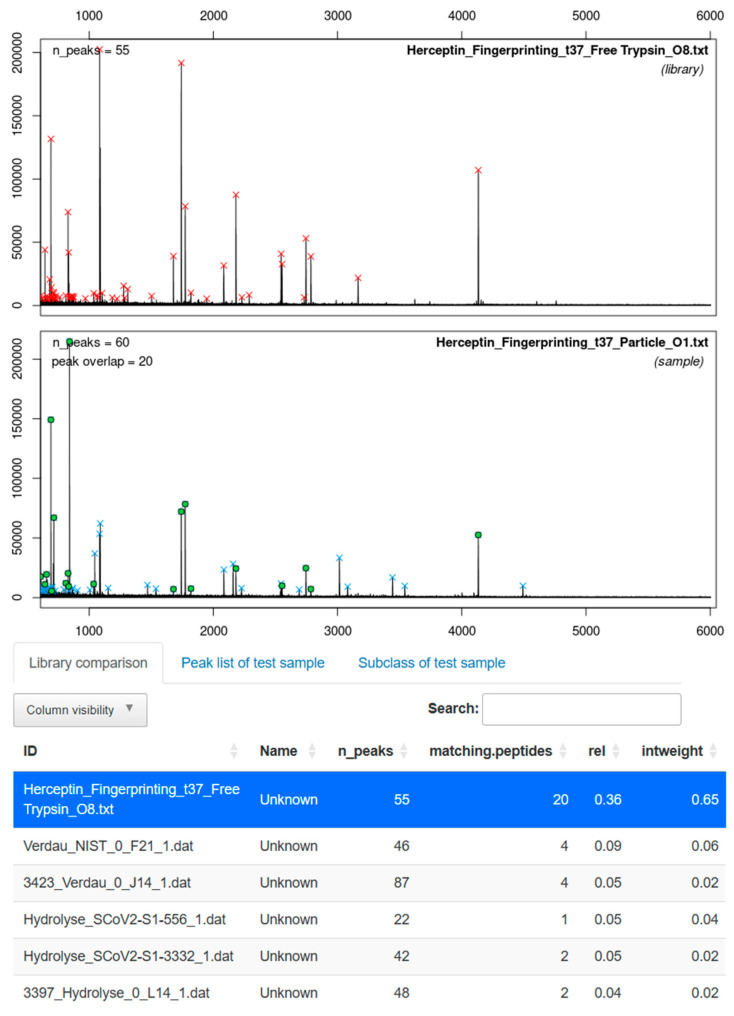
Comparison of MALDI-TOF MS spectra from digested Herceptin with trypsin-immobilized corundum particles (sample) and trypsin in solution (library) with ABID 2.0 software [64]. After digestion at 37 °C, the software finds 20 matching peptides corresponding to a score of 0.65. The next best matches only have ≤4 matching peptides leading to a score of ≤0.06. The red and blue crosses indicate the detected peaks, while the green points highlight the overlapping peaks with the reference spectrum from the library (highlighted in blue below).

The peptide fingerprints obtained with immobilized trypsin after digestion at 37 °C were correctly assigned to digested Herceptin by free trypsin with a 65% match score (Figure 7). Furthermore, at elevated temperatures of 60 °C, corundum-functionalized trypsin produces identifiable fingerprints with a match score of around 63% (Figure S8). In both cases, all other entries in the library scored below 6%. In addition, measured peptide masses were compared to theoretical values derived from in silico cleavage of the Herceptin heavy and light chain sequences. Overall, a sequence coverage of 60.3% was achieved for the antibody with digestion at 37 °C using corundum-functionalized trypsin (Figure S9).

## 4. Discussion

In this work, corundum was used to establish a new immobilization platform for enzymatic digestion of proteins with trypsin as a model proteolytic enzyme due to its central role in proteomics and peptide-based characterization.

The absence of production-related surface irregularities (Figure 1) indicates that the selected corundum material provided a better starting material compared to previously described particles [22]. Functionalization with APTES and glutaraldehyde was successfully verified by the Kaiser and Schiff tests, providing direct visual evidence of successful surface modification (Figure 2). The quantified amount of immobilized trypsin (approx. 1 µg per mg corundum) via AAAA was low compared to other materials such as agarose (50 µg/mg) [17], magnetic beads (30–60 µg/mg) [44,72] or chitosan (16 µg/mg) [48]. This can be attributed to the non-porous nature of corundum, which limits the available surface area. However, this drawback is compensated by its extremely low material cost (<0.30 €/g) compared to commercially available supports (>100 €/g), allowing the use of larger carrier quantities without economic limitations. For comparison, commercial immobilized trypsin products such as Mag-Trypsin™ cost approximately 450 USD per 30 mg and are typically used at around 300 µg of particles per 50 µg of protein, illustrating the substantial material demand and carrier-related cost of such systems.

SDS-PAGE analysis revealed strong non-specific protein adsorption for glutaraldehyde-modified particles, highlighting the need for blocking steps (Figure 3b). Using a blocking buffer consisting of Tris [73] and high concentrated amino acids glycine and lysine [74], residual aldehydes were effectively quenched, and non-specific protein binding was reduced. Moreover, faint residual bands observed when trypsin was immobilized suggest minor non-specific adsorption mediated by the enzyme itself due to protein–protein interactions. Furthermore, the absence of trypsin bands in the SDS eluates supports the conclusion that the enzyme was covalently immobilized and not only passively adsorbed onto the corundum surface.

Enzyme activity measurements with BAPNA revealed that trypsin-immobilized corundum particles achieved a specific activity of 0.13 U/mg and an approximately four-fold decrease compared to the free enzyme. This value falls within the expected range for immobilized enzyme systems, as reported for magnetic particles between 0.02 and 9 U/mg [72,75]. Such decrease is commonly attributed to steric hindrance and diffusion limitation commonly observed with other immobilized systems [44,45,76]. To minimize this effect in subsequent experiments, higher amounts of immobilized trypsin were used compared to free enzyme. While such an adjustment cannot fully compensate for diffusional and steric constraints, it ensured sufficient catalytic activity for downstream digestion and stability studies. The shift in the activity optimum from 50 °C for free trypsin to 60 °C for immobilized trypsin demonstrated increased robustness at elevated temperatures (Figure 4b). This effect can be attributed to enhanced conformational stability through immobilization and to the reduced extent of autocatalysis [46]. For instance, cellulose-coated or polyvinyl alcohol-coated magnetic nanoparticles have shown either increased relative activity at elevated temperatures (40 °C to 80 °C) or a shift in the activity optimum by approximately 10 °C toward higher temperatures [43,44]. In addition, high activity after incubation in denaturing reagents such as Gdn-HCl underlines the advantage of improved chaotropic resistance (Figure 5a).

In this study, the applicability of corundum-immobilized trypsin was demonstrated by antibody digestion and its suitability for peptide-based LC–MS/MS quantification as well as MALDI-TOF MS fingerprinting for antibody identification. In the LC–MS/MS measurements, immobilized trypsin enabled peptide-based quantification of the NISTmAb reference antibody comparable to free trypsin under standard conditions. Notably, immobilization provided an advantage in thermal tolerance at 60 °C compared to the free enzyme, although complete protein digestion was not achieved. This improved performance of immobilized enzyme may partly result from a reduced tendency of the immobilized enzyme toward self-digestion (autocleavage) and enhanced structural stability at elevated temperatures. In contrast, potential autolysis and thermal inactivation of free trypsin may have contributed to the loss of activity and lower peptide recovery observed at higher temperatures, although this was not explicitly investigated in the present study. Prolonged incubation at 60 °C may have further limited enzyme–substrate interactions due to particle sedimentation, thereby counteracting the initial kinetic advantage observed at elevated temperature. Consequently, 37 °C remained the most efficient condition for extended digestion. Based on the recovery rate, the quantitative results matched the concentrations specified in the NISTmAb certificate, demonstrating the applicability of the novel trypsin particles for accurate protein quantification [52,53].

Time-course experiments further indicated that short digests benefited from 50 °C incubation, whereas overnight protocols remained more efficient at 37 °C. This can be explained by gradual thermal inactivation and limited residual autolysis of the enzyme during extended incubation, even in its immobilized form [48,77]. In addition, sedimentation of corundum particles likely reduced enzyme–substrate contact over time, while peptide adsorption at elevated temperature may have further limited accessibility [78]. These combined effects progressively counteracted the initial kinetic advantage observed at 50 °C. Consequently, the enzyme maintained higher overall efficiency and stability under standard 37 °C conditions for prolonged incubations.

In parallel, MALDI-TOF MS fingerprinting demonstrated that corundum-immobilized trypsin can also generate peptide patterns suitable for antibody identification. Herceptin digests produced fingerprints that were unambiguously matched to the correct reference spectrum in a library of over 70 entries, even when digestion was performed at elevated temperature. Sequence coverage of 60.3% further supports that the immobilized enzyme provides sufficient cleavage efficiency for peptide mapping. Comparable results have been reported for functionalized magnetic particles, such as 62% for cytochrome c (12 kDa) [43], while higher coverages of 75 to 88% have been achieved for structurally less complex proteins such as BSA (66.5 kDa) compared to antibodies [53,72]. This highlighting the influence of substrate size and three-dimensional structure on achievable sequence coverage.

One of the key practical benefits of immobilization is the possibility of enzyme reuse. Substrate assays showed sustained activity above 80% over several cycles at 37 °C (Figure 5b). In antibody digestion experiments, efficiency remained stable over the first cycles and declined clearly after the fifth reuse. The gradual activity loss observed during repeated use likely results from partial enzyme deactivation. A minor carryover was observed, indicating that target peptide residues remained associated with the particles. Slight recovery values above 100% can be explained by this residual peptide carryover together with normal LC–MS/MS quantification variability, which can cause small deviations in peptide abundance estimates. Despite washing with 0.1% Tween 20, hydrophobic and electrostatic surface interactions with peptides likely prevented complete removal [79,80]. Previous studies have shown that supplementing the washing protocol with stronger detergents (e.g., 0.1% SDS) or organic solvents such as 40 to 80% acetonitrile or methanol effectively disrupts peptide adsorption without impairing trypsin activity [52,80,81]. Incorporating such washing steps could further minimize carryover and enhance reusability in repeated digestion workflows, and where appropriate subtracting the ALPAPIEK blank run signal as a background correction can further improve quantitative accuracy.

The comparison of BAPNA and LC–MS/MS readouts highlights that catalytic activity decreases faster than digestion efficiency, which may reflect differences in sensitivity between different substrates. Small molecules such as BAPNA readily access the active site and therefore respond more sensitively to partial enzyme inactivation, whereas protein hydrolysis in immobilized systems is often diffusion-limited [1,3,19,43]. Because the corundum support is non-porous, internal diffusion barriers are minimal, yet external mass-transfer effects such as particle sedimentation and reduced substrate contact can still influence digestion efficiency. As a consequence, complex protein digestion can still proceed efficiently over extended incubation times due to multiple cleavage sites and prolonged enzyme–substrate interaction. Similar observations have been reported for other immobilized trypsin systems, where reduced BAPNA activity did not proportionally affect protein digestion performance [82,83,84]. Extended incubation times further compensate for moderate activity losses, ensuring sufficient proteolysis even after several reuse cycles [84,85].

## 5. Conclusions

Together, the results show that covalent immobilization of trypsin to corundum particles not only enhances tolerance under thermal and denaturing conditions through structural stabilization but also enables repeated use of the enzyme. These features underline its suitability and practical as well as economic benefits for robust and scalable protein digestion workflows.

The demonstrated use of corundum-immobilized trypsin in both antibody quantification and identification provides a strong foundation for broader analytical deployment. Evaluating its performance in complex biological matrices such as serum or cell lysates and assessing its compatibility with high-throughput MS workflows would further highlight the versatility and translational potential of corundum-immobilized enzymes in modern proteomics. Expanding the enzymatic portfolio beyond trypsin represents another opportunity for application. Sequential or co-immobilization of complementary proteases (e.g., LysC, Pepsin) has been shown to improve sequence coverage and digestion speed [86].

Future developments could focus on refining enzyme orientation and surface architecture. Recent studies have demonstrated that spatial control via DNA scaffold structures can reduce steric hindrance and enhance enzymatic accessibility [53]. Integrating such directed immobilization strategies with the corundum platform may further improve catalytic efficiency and facilitate reusing. In this context, microenvironmental effects such as enzyme orientation or conformational constraints may also influence catalytic performance and represent an important direction for future investigations. In addition to improved elution and washing protocols, alternative surface chemistries may further reduce non-specific protein interactions. Silane variants such as (3-triethoxysilyl)butyraldehyde (TESB) or coatings like hyperbranched polyglycerol have shown potential to enhance coupling efficiency and minimize non-specific binding [15,22]. Furthermore, replacing toxic crosslinkers such as glutaraldehyde with greener alternatives could improve the environmental and operational safety profile of the immobilization process, which is particularly relevant for industrial applications.

A further promising next step involves integrating of corundum-based carriers into microreactor systems [87]. Additively manufactured or 3D-printed microcolumns combined with immobilized enzyme beads have already shown significantly accelerated and more complete digestions [88]. Combining the mechanical robustness of corundum with the precision of microfabrication could yield durable, high-performance enzyme reactors suitable for continuous operation.

In summary, combining oriented immobilization chemistry, advanced immobilization strategies by increased surface area, and microreactor integration could further unlock the potential of corundum-based enzyme carriers for diverse analytical and industrial applications.

## Data Availability

The sample overview and extracted data of LC-MS/MS measurements are available in Supplementary Materials. The raw data of LC-MS/MS measurements and MALDI-TOF MS are available on Zenodo (doi:10.5281/zenodo.17416537, accessed on 19 November 2025).

## Supplementary Materials

The following supporting information can be downloaded at: https://www.mdpi.com/article/10.3390/biotech15010002/s1. Method: Recombinant production of porcine trypsin. Table S1: Q1 isolation window for SWATH acquisition mode used for LC-MS/MS measurement of NISTmAb tryptic peptides. The accumulation (dwell) time was 0.05 s per window. Collision energy (CE) varied between 10 eV (window 1) and 67 eV (window 11). A collision energy spread of 15 eV was used in all windows; Figure S1: Particle size determination of corundum F1500 in ultrapure water indicated a particle size of around 2 µm (D50); Figure S2: Dynamic light scattering (DLS) measurements of raw corundum F1500 (1.55 µm) and after trypsin immobilization (1.58 µm) in Ultrapure water; Figure S3: TEM-EDS analysis of raw corundum F1500; Table S2: Results of Kaiser test. The absorbance (OD) of the Ruhemann’s purple was measured for raw corundum (C), APTES-silanized corundum (CA) and glutaraldehyde-modified corundum (CAG) photometrically at 586 nm; Figure S4: Measurement of calibration tyrosine standards (**a**) and determination of external calibration curve based on peak area (**b**); Figure S5: Storage stability of trypsin-functionalized corundum particles for one or 4 weeks at 22 °C, 4 °C and −20 °C; Figure S6: Recovery rate of NISTmAb via surrogate peptide ALPAPIEK after digestion with trypsin-functionalized particles at different time points and different temperatures; Table S3: Overview of identified heavy chain peptides of digested NISTmAb by trypsin-immobilized corundum particles; Table S4: Overview of identified light chain peptides of digested NISTmAb by trypsin-immobilized corundum particles; Table S5: Overview of identified heavy chain peptides of digested NISTmAb with trypsin in solution; Table S6: Overview of identified heavy chain peptides of digested NISTmAb with trypsin in solution; Figure S7: Reusability of immobilized trypsin particles over consecutive digests at 37 °C, monitored recovery rate by LC–MS/MS (blue) and residual enzyme activity in the BAPNA assay (red) including blank runs without antibody (number 2 and 4); Figure S8: Comparison of MALDI-TOF MS spectra from digested Herceptin at 60 °C with trypsin-immobilized corundum particles (sample) and trypsin in solution (library) with ABID 2.0 software; Figure S9: MALDI-TOF fingerprint spectrum of Herceptin after 15 min of incubation with TCEP at 99 °C, followed by 60 min of incubation with trypsin-functionalized particles at 37 °C; Sample overview and extracted raw data files of LC-MS/MS measurement; References [63,64] are cited in Supplementary Materials.

## References

[1] Cavalcante F.T., Cavalcante A.L., de Sousa I.G., Neto F.S., dos Santos J.C. (2021). Current status and future perspectives of supports and protocols for enzyme immobilization. Catalysts.

[2] Bié J., Sepodes B., Fernandes P.C., Ribeiro M.H. (2022). Enzyme immobilization and co-immobilization: Main framework, advances and some applications. Processes.

[3] Guisan J.M., Fernandez-Lorente G., Rocha-Martin J., Moreno-Gamero D. (2022). Enzyme immobilization strategies for the design of robust and efficient biocatalysts. Curr. Opin. Green Sustain. Chem..

[4] Maghraby Y.R., El-Shabasy R.M., Ibrahim A.H., Azzazy H.M.E.-S. (2023). Enzyme immobilization technologies and industrial applications. ACS Omega.

[5] Homaei A.A., Sariri R., Vianello F., Stevanato R. (2013). Enzyme immobilization: An update. J. Chem. Biol..

[6] Budžaki S., Miljić G., Sundaram S., Tišma M., Hessel V. (2018). Cost analysis of enzymatic biodiesel production in small-scaled packed-bed reactors. Appl. Energy.

[7] Federsel H.-J., Moody T.S., Taylor S.J. (2021). Recent trends in enzyme immobilization—Concepts for expanding the biocatalysis toolbox. Molecules.

[8] Asanomi Y., Yamaguchi H., Miyazaki M., Maeda H. (2011). Enzyme-immobilized microfluidic process reactors. Molecules.

[9] DiCosimo R., McAuliffe J., Poulose A.J., Bohlmann G. (2013). Industrial use of immobilized enzymes. Chem. Soc. Rev..

[10] Liese A., Hilterhaus L. (2013). Evaluation of immobilized enzymes for industrial applications. Chem. Soc. Rev..

[11] Lee H., Hong Y.J., Baik S., Hyeon T., Kim D.H. (2018). Enzyme-based glucose sensor: From invasive to wearable device. Adv. Healthc. Mater..

[12] Nguyen H.H., Lee S.H., Lee U.J., Fermin C.D., Kim M. (2019). Immobilized enzymes in biosensor applications. Materials.

[13] Naldi M., Tramarin A., Bartolini M. (2018). Immobilized enzyme-based analytical tools in the-omics era: Recent advances. J. Pharm. Biomed. Anal..

[14] Nagy C., Szabo R., Gaspar A. (2022). Microfluidic immobilized enzymatic reactors for proteomic analyses—Recent developments and trends (2017–2021). Micromachines.

[15] Yao Z., Li Y., Xu W. (2025). Micro-immobilized enzyme reactors for mass spectrometry proteomics. Analyst.

[16] Basso A., Froment L., Hesseler M., Serban S. (2013). New highly robust divinyl benzene/acrylate polymer for immobilization of lipase CALB. Eur. J. Lipid Sci. Technol..

[17] dos Santos J.C., Rueda N., Barbosa O., del Carmen Millán-Linares M., Pedroche J., del Mar Yuste M., Goncalves L.R., Fernandez-Lafuente R. (2015). Bovine trypsin immobilization on agarose activated with divinylsulfone: Improved activity and stability via multipoint covalent attachment. J. Mol. Catal. B Enzym..

[18] Lavlinskaya M.S., Sorokin A.V., Holyavka M.G., Zuev Y.F., Artyukhov V.G. (2025). Cellulose and cellulose-based materials for enzyme immobilization: A review. Biophys. Rev..

[19] Hartmann M., Kostrov X. (2013). Immobilization of enzymes on porous silicas–benefits and challenges. Chem. Soc. Rev..

[20] Zhao L., Zhang Y., Yang Y., Yu C. (2022). Silica-based nanoparticles for enzyme immobilization and delivery. Chem. Asian J..

[21] Ertesvåg H. (2015). Alginate-modifying enzymes: Biological roles and biotechnological uses. Front. Microbiol..

[22] Völzke J.L., Shamami P.H., Gawlitza K., Feldmann I., Zimathies A., Meyer K., Weller M.G. (2022). High-purity corundum as support for affinity extractions from complex samples. Separations.

[23] Völzke J.L., Smatty S., Döring S., Ewald S., Oelze M., Fratzke F., Flemig S., Konthur Z., Weller M.G. (2023). Efficient Purification of Polyhistidine-Tagged Recombinant Proteins Using Functionalized Corundum Particles. BioTech.

[24] Vashist S.K., Lam E., Hrapovic S., Male K.B., Luong J.H. (2014). Immobilization of antibodies and enzymes on 3-aminopropyltriethoxysilane-functionalized bioanalytical platforms for biosensors and diagnostics. Chem. Rev..

[25] Sypabekova M., Hagemann A., Rho D., Kim S. (2022). 3-Aminopropyltriethoxysilane (APTES) deposition methods on oxide surfaces in solution and vapor phases for biosensing applications. Biosensors.

[26] Migneault I., Dartiguenave C., Bertrand M.J., Waldron K.C. (2004). Glutaraldehyde: Behavior in aqueous solution, reaction with proteins, and application to enzyme crosslinking. Biotechniques.

[27] Barbosa O., Ortiz C., Berenguer-Murcia Á., Torres R., Rodrigues R.C., Fernandez-Lafuente R. (2014). Glutaraldehyde in bio-catalysts design: A useful crosslinker and a versatile tool in enzyme immobilization. RSC Adv..

[28] Clinton F. (1975). Sodium cyanoborohydride—A highly selective reducing agent for organic functional groups. Synthesis.

[29] Germain P., Slagmolen T., Crichton R. (1989). Relation between stabilization and rigidification of the three-dimensional structure of an enzyme. Biotechnol. Bioeng..

[30] Aebersold R., Mann M. (2003). Mass spectrometry-based proteomics. Nature.

[31] Shevchenko A., Tomas H., Havli J., Olsen J.V., Mann M. (2006). In-gel digestion for mass spectrometric characterization of proteins and proteomes. Nat. Protoc..

[32] Tsiatsiani L., Heck A.J. (2015). Proteomics beyond trypsin. FEBS J..

[33] Sinha A., Mann M. (2020). A beginner’s guide to mass spectrometry–based proteomics. Biochemist.

[34] Noor Z., Ahn S.B., Baker M.S., Ranganathan S., Mohamedali A. (2021). Mass spectrometry–based protein identification in proteomics—A review. Brief. Bioinform..

[35] Döring S., Weller M.G., Reinders Y., Konthur Z., Jaeger C. (2025). Challenges and insights in absolute quantification of recombinant therapeutic antibodies by mass spectrometry: An introductory review. Antibodies.

[36] Furlong M.T., Ouyang Z., Wu S., Tamura J., Olah T., Tymiak A., Jemal M. (2012). A universal surrogate peptide to enable LC-MS/MS bioanalysis of a diversity of human monoclonal antibody and human Fc-fusion protein drug candidates in pre-clinical animal studies. Biomed. Chromatogr..

[37] Zhang S., Jian W. (2014). Recent advances in absolute quantification of peptides and proteins using LC-MS. Rev. Anal. Chem..

[38] Pappin D.J., Hojrup P., Bleasby A.J. (1993). Rapid identification of proteins by peptide-mass fingerprinting. Curr. Biol..

[39] Pappin D.J. (2003). Peptide mass fingerprinting using MALDI-TOF mass spectrometry. Protein Sequencing Protocols.

[40] Wu F., Zhao M., Zhang Y., Su N., Xiong Z., Xu P. (2016). Recombinant acetylated trypsin demonstrates superior stability and higher activity than commercial products in quantitative proteomics studies. Rapid Commun. Mass Spectrom..

[41] Muriithi B., Ippoliti S., Finny A., Addepalli B., Lauber M. (2024). Clean and complete protein digestion with an autolysis resistant trypsin for peptide mapping. J. Proteome Res..

[42] Menneteau T., Saveliev S., Butré C.I., Rivera A.K.G., Urh M., Delobel A. (2024). Addressing common challenges of biotherapeutic protein peptide mapping using recombinant trypsin. J. Pharm. Biomed. Anal..

[43] Sun X., Cai X., Wang R.-Q., Xiao J. (2015). Immobilized trypsin on hydrophobic cellulose decorated nanoparticles shows good stability and reusability for protein digestion. Anal. Biochem..

[44] Sahin S., Ozmen I. (2020). Covalent immobilization of trypsin on polyvinyl alcohol-coated magnetic nanoparticles activated with glutaraldehyde. J. Pharm. Biomed. Anal..

[45] Aversa I.F., Cavalcanti M.H., Pereira T.M., A. de Castro A., Tavano O.L., Coelho Y.L., da Silva L.H., Gorup L.F., Ramalho T.C., Virtuoso L.S. (2025). Immobilization of Porcine Trypsin in Superparamagnetic Nanoparticles: Enzyme Activity and Stability. ACS Omega.

[46] Aslani E., Abri A., Pazhang M. (2018). Immobilization of trypsin onto Fe_3_O_4_@ SiO_2_–NH_2_ and study of its activity and stability. Colloids Surf. B Biointerfaces.

[47] Kim J.S., Lee S. (2019). Immobilization of trypsin from porcine pancreas onto chitosan nonwoven by covalent bonding. Polymers.

[48] Miguez J.P., Fernandez-Lafuente R., Tavano O.L., Mendes A.A. (2023). The immobilization and stabilization of trypsin from the porcine pancreas on chitosan and its catalytic performance in protein hydrolysis. Catalysts.

[49] Massolini G., Calleri E. (2005). Immobilized trypsin systems coupled on-line to separation methods: Recent developments and analytical applications. J. Sep. Sci..

[50] Regnier F.E., Kim J. (2014). Accelerating trypsin digestion: The immobilized enzyme reactor. Bioanalysis.

[51] Naldi M., Černigoj U., Štrancar A., Bartolini M. (2017). Towards automation in protein digestion: Development of a monolithic trypsin immobilized reactor for highly efficient on-line digestion and analysis. Talanta.

[52] Reinders L.M., Klassen M.D., Teutenberg T., Jaeger M., Schmidt T.C. (2021). Development of a multidimensional online method for the characterization and quantification of monoclonal antibodies using immobilized flow-through enzyme reactors. Anal. Bioanal. Chem..

[53] Fan X., Chu Z., Zhu M., Song Y., Zhao Y., Meng B., Gong X., Zhang D., Jiang Y., Wu L. (2023). Precise control of trypsin immobilization by a programmable DNA tetrahedron designed for ultrafast proteome digestion and accurate protein quantification. Anal. Chem..

[54] Tscheuschner G., Schwaar T., Weller M.G. (2020). Fast confirmation of antibody identity by MALDI-TOF MS fingerprints. Antibodies.

[55] Tscheuschner G., Kaiser M.N., Lisec J., Beslic D., Muth T., Krüger M., Mages H.W., Dorner B.G., Knospe J., Schenk J.A. (2022). MALDI-TOF-MS-based identification of monoclonal murine anti-SARS-CoV-2 antibodies within one hour. Antibodies.

[56] Schiel J.E., Turner A. (2018). The NISTmAb Reference Material 8671 lifecycle management and quality plan. Anal. Bioanal. Chem..

[57] Kaiser E., Colescott R.L., Bossinger C.D., Cook P. (1970). Color test for detection of free terminal amino groups in the solid-phase synthesis of peptides. Anal. Biochem..

[58] Sarin V.K., Kent S.B., Tam J.P., Merrifield R.B. (1981). Quantitative monitoring of solid-phase peptide synthesis by the ninhydrin reaction. Anal. Biochem..

[59] Stauß A.C., Fuchs C., Jansen P., Repert S., Alcock K., Ludewig S., Rozhon W. (2024). The ninhydrin reaction revisited: Optimisation and application for quantification of free amino acids. Molecules.

[60] Reinmuth-Selzle K., Tchipilov T., Backes A.T., Tscheuschner G., Tang K., Ziegler K., Lucas K., Pöschl U., Fröhlich-Nowoisky J., Weller M.G. (2022). Determination of the protein content of complex samples by aromatic amino acid analysis, liquid chromatography-UV absorbance, and colorimetry. Anal. Bioanal. Chem..

[61] Erlanger B.F., Kokowsky N., Cohen W. (1961). The preparation and properties of two new chromogenic substrates of trypsin. Arch. Biochem. Biophys..

[62] Tsugawa H., Cajka T., Kind T., Ma Y., Higgins B., Ikeda K., Kanazawa M., VanderGheynst J., Fiehn O., Arita M. (2015). MS-DIAL: Data-independent MS/MS deconvolution for comprehensive metabolome analysis. Nat. Methods.

[63] Dong Q., Liang Y., Yan X., Markey S.P., Mirokhin Y.A., Tchekhovskoi D.V., Bukhari T.H., Stein S.E. (2018). The NISTmAb tryptic peptide spectral library for monoclonal antibody characterization. MAbs.

[64] Lisec J. ABID. https://github.com/BAMresearch/ABID.

[65] Schiff H. (1866). Eine neue reihe organischer diamine. Justus Liebigs Ann. Der Chem..

[66] Wieland H., Scheuing G. (1921). Die Fuchsin-schweflige Säure und ihre Farbreaktion mit Aldehyden. Berichte Der Dtsch. Chem. Ges. (A B Ser.).

[67] Hesse A., Weller M.G. (2016). Protein quantification by derivatization-free high-performance liquid chromatography of aromatic amino acids. J. Amino Acids.

[68] Bronsema K.J., Bischoff R., Pijnappel W.P., van der Ploeg A.T., Van De Merbel N.C. (2015). Absolute quantification of the total and antidrug antibody-bound concentrations of recombinant human α-glucosidase in human plasma using protein G extraction and LC-MS/MS. Anal. Chem..

[69] Mi W., Josephs R., Melanson J., Dai X., Wang Y., Zhai R., Chu Z., Fang X., Thibeault M., Stocks B. (2022). PAWG pilot study on quantification of SARS-CoV-2 monoclonal antibody-part 1. Metrologia.

[70] Martos G., Bedu M., Josephs R., Westwood S., Wielgosz R. (2024). Quantification of SARS-CoV-2 monoclonal IgG mass fraction by isotope dilution mass spectrometry. Anal. Bioanal. Chem..

[71] Harries M., Smith I. (2002). The development and clinical use of trastuzumab (Herceptin). Endocr. Relat. Cancer.

[72] Lee B., Lopez-Ferrer D., Kim B.C., Na H.B., Park Y.I., Weitz K.K., Warner M.G., Hyeon T., Lee S.W., Smith R.D. (2011). Rapid and efficient protein digestion using trypsin-coated magnetic nanoparticles under pressure cycles. Proteomics.

[73] Bubb W.A., Berthon H.A., Kuchel P.W. (1995). Tris buffer reactivity with low-molecular-weight aldehydes: NMR characterization of the reactions of glyceraldehyde-3-phosphate. Bioorganic Chem..

[74] Kim K.S., Lee Y., Lee J.H., Lee S.S., Chung J.M., Jung H.S. (2024). Optimizing protein crosslinking control: Synergistic quenching effects of glycine, histidine, and lysine on glutaraldehyde reactions. Biochem. Biophys. Res. Commun..

[75] Maciel J., Andrad P., Neri D., Carvalho L., Cardoso C., Calazans G., Aguiar J.A., Silva M. (2012). Preparation and characterization of magnetic levan particles as matrix for trypsin immobilization. J. Magn. Magn. Mater..

[76] Doğan D., Sezer S., Ulu A., Köytepe S., Ateş B. (2021). Preparation and characterization of amino-functionalized zeolite/SiO2 materials for trypsin–chymotrypsin co-immobilization. Catal. Lett..

[77] Sanchez A., Cruz J., Rueda N., dos Santos J.C., Torres R., Ortiz C., Villalonga R., Fernandez-Lafuente R. (2016). Inactivation of immobilized trypsin under dissimilar conditions produces trypsin molecules with different structures. RSC Adv..

[78] Sun L., Zhu G., Yan X., Mou S., Dovichi N.J. (2014). Uncovering immobilized trypsin digestion features from large-scale proteome data generated by high-resolution mass spectrometry. J. Chromatogr. A.

[79] Yuan H., Zhang L., Zhang Y. (2014). Preparation of high efficiency and low carry-over immobilized enzymatic reactor with methacrylic acid–silica hybrid monolith as matrix for on-line protein digestion. J. Chromatogr. A.

[80] Kumar S., Anderson K.W. (2024). Rapid removal of IgG1 carryover on protease column using protease-safe wash solutions delivered with LC pump for HDX-MS systems. J. Am. Soc. Mass Spectrom..

[81] Liang Y., Tao D., Ma J., Sun L., Liang Z., Zhang L., Zhang Y. (2011). Hydrophilic monolith based immobilized enzyme reactors in capillary and on microchip for high-throughput proteomic analysis. J. Chromatogr. A.

[82] Kim B.C., Lopez-Ferrer D., Lee S.M., Ahn H.K., Nair S., Kim S.H., Kim B.S., Petritis K., Camp D.G., Grate J.W. (2009). Highly stable trypsin-aggregate coatings on polymer nanofibers for repeated protein digestion. Proteomics.

[83] Camperi J., Grunert I., Heinrich K., Winter M., Özipek S., Hoelterhoff S., Weindl T., Mayr K., Bulau P., Meier M. (2021). Inter-laboratory study to evaluate the performance of automated online characterization of antibody charge variants by multi-dimensional LC-MS/MS. Talanta.

[84] Kaeek M., Khoury L.R. (2025). Bovine Serum Albumin–Trypsin Sponges for Enhanced Enzymatic Stability and Protein Digestion Efficiency. ACS Appl. Bio Mater..

[85] Oezipek S., Hoelterhoff S., Breuer S., Bell C., Bathke A. (2022). mD-UPLC-MS/MS: Next generation of mAb characterization by multidimensional ultraperformance liquid chromatography-mass spectrometry and parallel on-column lysC and trypsin digestion. Anal. Chem..

[86] Yuan F.-F., Wang P., Han X.-J., Qin T.-T., Lu X., Bai H.-J. (2024). Efficient and rapid digestion of proteins with a dual-enzyme microreactor featuring 3-D pores formed by dopamine/polyethyleneimine/acrylamide-coated KIT-6 molecular sieve. Sci. Rep..

[87] Wilke M., Röder B., Paul M., Weller M.G. (2021). Sintered glass monoliths as supports for affinity columns. Separations.

[88] Rainer T., Egger A.-S., Zeindl R., Tollinger M., Kwiatkowski M., Müller T. (2022). 3D-printed high-pressure-resistant immobilized enzyme microreactor (ΜIMER) for protein analysis. Anal. Chem..

