# Supplementary material for: Corundum Particles as Trypsin Carrier for Efficient Protein Digestion
Source: BioTech (Basel). 2025 Dec 30;15(1):2. doi: 10.3390/biotech15010002 (PMC12821454; doi:10.3390/biotech15010002)
Supplement: Supplementary file 1 [file biotech-15-00002-s001.zip › Supplementary Material.pdf]

BioTech

MDPI

Supplementary materials

Corundum Particles as Trypsin Carrier for Efficient Protein Digestion

Sarah Döring[1], Birte S. Wulfes[1], Aleksandra Atanasova[1], Carsten Jaeger[1], Leopold Walzel, Georg Tscheuschner[1,2], Sabine Flemig[1], Kornelia Gawlitza[1], Ines Feldmann[1], Zoltán Konthur[1] and Michael G. Weller[1*]

1 Federal Institute for Materials Research and Testing (BAM), Richard-Willstätter-Strasse 11, 12489 Berlin, Germany; sarah.doering@bam.de (S.D.); carsten.jaeger@bam.de (C.J.); leopold.walzel@bam.de (L.W.); tscheuschnerg@rki.de (G.T.); sabine.flemig@bam.de (S.F.); kornelia.gawlitza@bam.de (K.G.); ines.feldmann@bam.de (I.F.); zoltan.konthur@bam.de (Z.K.)

2 Biological Toxins, Centre for Biological Threats and Special Pathogens, Robert Koch Institute, Seestraße 10, 13353 Berlin, Germany

* Correspondence: michael.weller@bam.de

Recombinant production of porcine trypsin

The amino acid sequence of porcine trypsin (UniProt ID: P00761) was obtained from the National Center of Biotechnology Information (NCBI). Six histidine residues with a propeptide sequence (MGSAHHHHHH ASSGFPTDDDDK) were added to the N-terminal, and an Avidin-Tag (GLNDIFEAQKIEWHE) was added to the C-terminal of trypsin. The codon-optimized sequence was synthesized by GENEWIZ. For cloning, the synthesized sequence and pET vector were digested with NdeI and XhoI. The fragments were purified from agarose gels and ligated with T4 DNA ligase (New England Biolabs, Ipswich, MA, USA). The constructed vector was verified by sequencing (LGC Genomics, Teddington, UK).

The generated plasmid (pET-His6-Trypsinogen-3xGSA-AviTag) was transformed into the expression strain *E. coli* NiCo21(DE3) (New England Biolabs) via heat shock and selected on LB agar plates containing 30 µg/mL kanamycin and 2% glucose. Positive clones were cultured in LB medium with the same additives overnight at 200 rpm and 37 °C. Pre-warmed TB medium (17 mM KH_2PO_4, 72 mM K_2HPO_4, 12 g/L tryptone, 24 g/L yeast extract, 4% (v/v) glycerol) containing 30 µg/mL kanamycin was inoculated 1:100 with overnight culture and grown at 200 rpm and 37 °C until OD600 reached 0.6. Protein expression was induced with 0.1 mM IPTG for 4 h. After centrifugation at 10,000× *g* and 4 °C for 10 min, the pellet was resuspended in B-PER™ Complete Bacterial Protein Extraction Reagent (Thermo Fisher Scientific, Waltham, MA, USA) and incubated for 30 min in an overhead rotator at 30 rpm. Cells were then disrupted by three cycles of 10 s sonication with 10 s cooling intervals (QSonica Q125, Newtown, CT, USA). The lysate was centrifuged at 10,000× *g* and 4 °C for 20 min. The supernatant was discarded, and the pellet resuspended in denaturing lysis buffer (8 M urea, 100 mM sodium phosphate, 10 mM Tris, pH 8.0). After 30 min incubation at RT, the solution was centrifuged, and the supernatant was subjected to Ni-NTA purification (HisTrap 1 mL, Cytiva, Marlborough, MA, USA) with 8 M urea in the binding and elution buffer according to manufacturer instruction.

The trypsinogen eluate was diluted to 0.2 mg/mL and incubated with 20 mM DTT for 60 min at RT. Refolding was performed by stepwise dialysis for 24 h at 4 °C in Slide-A-Lyzer™ 3.5K Dialysis Cassettes (Thermo Fisher Scientific) against refolding buffer A (4 M urea, 20 mM sodium phosphate, 0.5 M NaCl, pH 8.0), buffer B (2 M urea, 100 mM Tris-HCl, 0.5 M L-arginine, 2 mM $CaCl_2$, 5 mM cysteine, 0.5 mM cystine, pH 8.0), buffer C (100 mM Tris-HCl, 0.5 M L-arginine, 2 mM $CaCl_2$, 5 mM cysteine, 0.5 mM cystine, pH 8.0) and finally enterokinase activation buffer (20 mM Tris-HCl, 50 mM NaCl, 2 mM $CaCl_2$, pH 7.4). The solution was then concentrated to 1 mg/mL using µPulse® Systems (For-mulatrix, Bedford, MA, USA) with a 5 kDa membrane. Enterokinase (0.2 µL per 50 µg trypsinogen; Z03004, GenScript, Piscataway Township, NJ, USA) was added and incubated overnight at RT and 300 rpm in a ThermoMixer C. On the following day, the propeptide and enterokinase were removed by incubation with 100 µL slurry Ni-NTA-agarose beads (Jena Biosciences, Jena, Germany) for 1 h at 4 °C and 30 rpm in overhead rotator. After centrifugation at 10,000× *g* and 4 °C for 20 min, the supernatant was filtered through 0.22 µm sterile filter and used for benzamidine affinity purification

(HiTrap™ Benzamidine FF, Cytiva) according to manufacturer instruction. The eluted fractions were collected in a volume of 500 µL, neutralized to pH 7.4 with 50 µL neutralization buffer (10x PBS, pH 8.5) and stored at −20 °C until use.

SWATH isolation windows

Table S1: Q1 isolation window for SWATH acquisition mode used for LC-MS/MS measurement of NISTmAb tryptic peptides. The accumulation (dwell) time was 0.05 s per window. Collision energy (CE) varied between 10 eV (window 1) and 67 eV (window 11). A collision energy spread of 15 eV was used in all windows.

Window no.	Start	End
1	200.00	290.91
2	289.91	381.82
3	380.82	472.73
4	471.73	563.64
5	562.64	654.55
6	653.55	745.45
7	744.45	836.36
8	835.36	927.27
9	926.27	1018.18
10	1017.18	1109.09
11	1108.09	1200.00

Particle size distribution of corundum

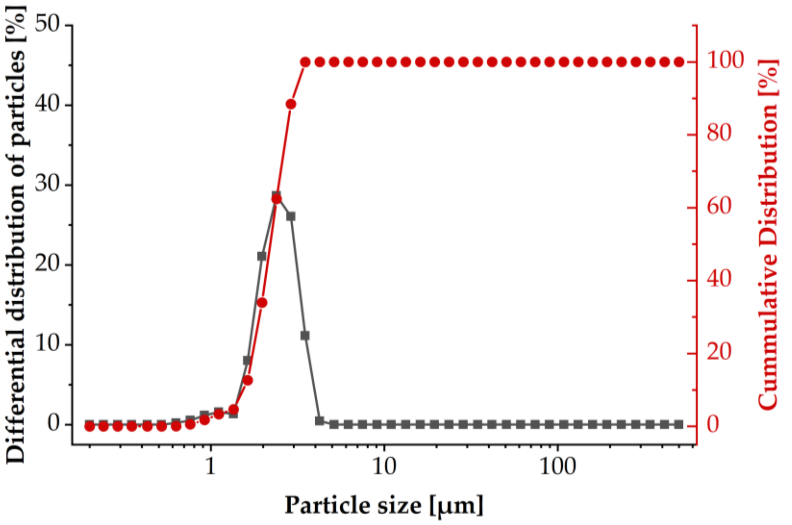

Figure S1: Particle size determination of corundum F1500 in ultrapure water indicated a particle size of around 2 µm (D50). The measurement was performed by the manufacturer (Haixu Abrasives, ZhengZhou, China).

Dynamic light scattering (DLS) measurements of corundum

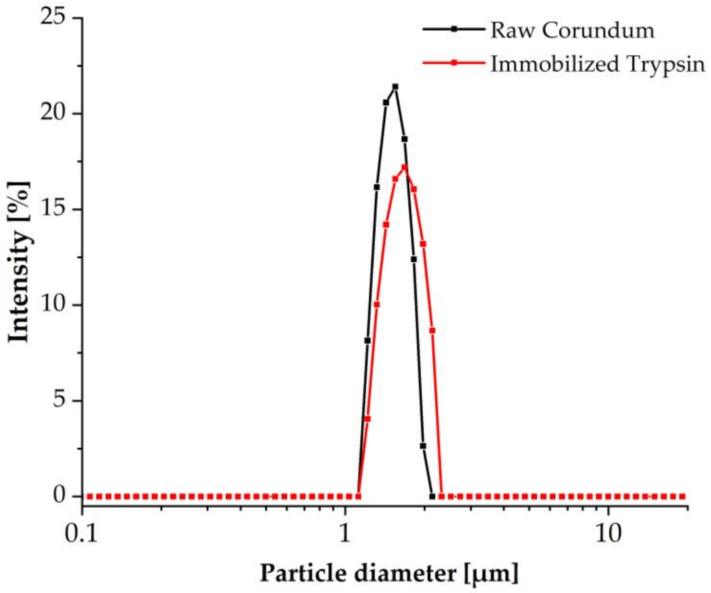

Figure S2: Dynamic light scattering (DLS) measurements of raw corundum F1500 (1.55 µm) and after trypsin immobilization (1.58 µm) in ultrapure water.

Energy-dispersive x-ray spectroscopy (EDS) of corundum

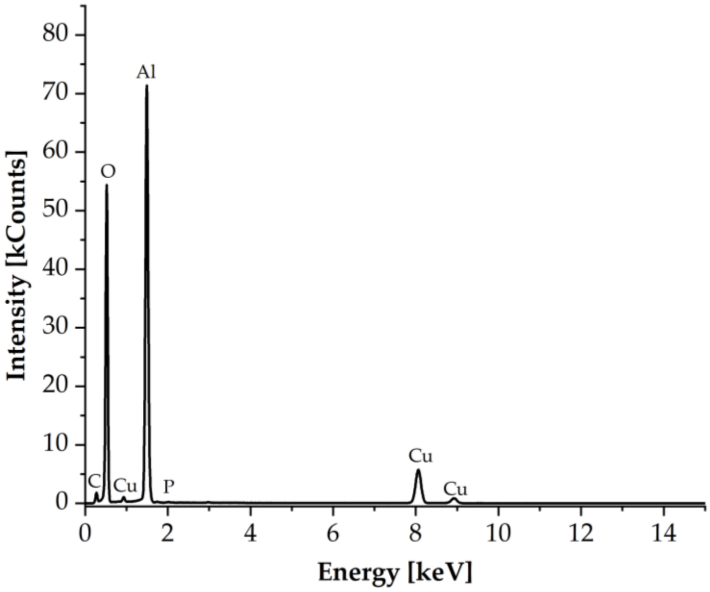

Figure S3: TEM-EDS analysis of raw corundum F1500.

Kaiser test of functionalized corundum

Table S2: Results of Kaiser test. The absorbance (OD) of the Ruhemann's purple was measured for raw corundum (C), APTES-silanized corundum (CA) and glutaraldehyde-modified corundum (CAG) photometrically at 586 nm.

Sample	Measured $OD_{586\ nm}$	Dilution Factor	Total $OD_{586\ nm}$	Availability of Free Amines [%]
C	0.006	1	0.006	0.24
CA	0.645	4	2.580	100.00
CAG	0.053	1	0.053	2.05

The percentage availability of free amines was calculated based on the assumption that, after salinization with APTES, all possible amino groups are accessible according to equation (1).

$$\text{Free Amines [\%]} = \frac{OD_{586\ nm}\text{of sample}}{OD_{586nm}\text{of CAG}} \times 100 \quad (1)$$

External calibration curve of tyrosine

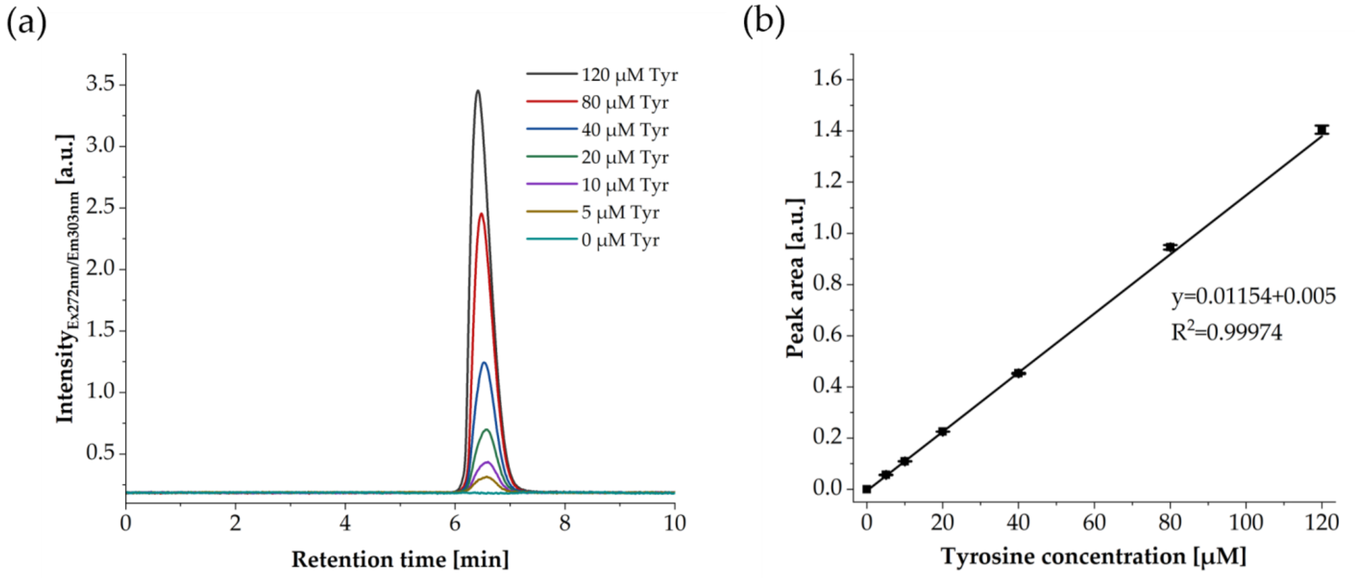

Figure S4: Measurement of calibration tyrosine standards (a) and determination of external calibration curve based on peak area (b). Fluorescence detection was performed at 272 nm (Ex.) and 303 nm (Em.). Errors were calculated as the standard deviation of duplicate measurements.

Storage stability of trypsin-immobilized corundum

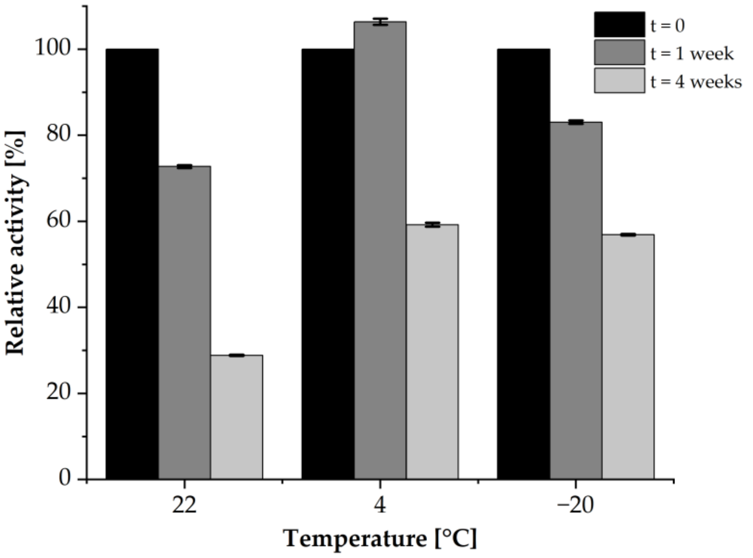

Figure S5: Storage stability of trypsin-functionalized corundum particles for one or 4 weeks at 22 °C, 4°C and -20 °C. Relative activity was calculated after incubation of 10 mg particles in 500 µM BAPNA substrate in 25 mM Tris (pH 8.0) for 15 min. Errors were calculated as the standard deviation of duplicate measurements.

Different digestion times for LC-MS/MS-based Quantification of NISTmAb

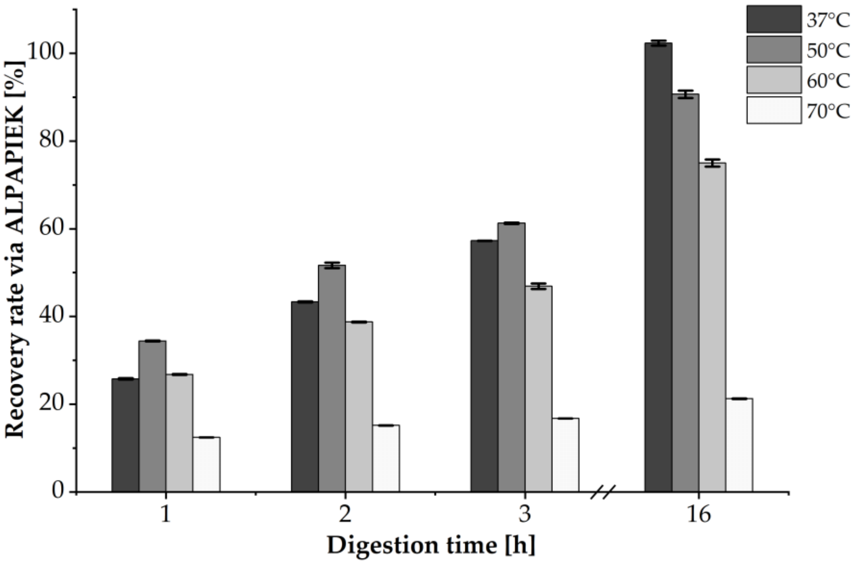

Figure S6: Recovery rate of NISTmAb via the peptide ALPAPIEK after digestion with trypsin-functionalized particles at different time points and different temperatures. The Recovery rate was calculated as the percentage of the measured peptide amount relative to the theoretical yield based on the known antibody input. Errors were calculated as the standard deviation of duplicate measurements.

Sequence Coverage of LC-MS/MS measured NISTmAb peptides after tryptic digestion

The assignment was performed using the spectral library of NISTmAb [63] with an identification score of 60 % for the fragments in MS2 spectra.

The NISTmAb spectral library can be downloaded at the website of Mass Spectrometry Data Center of NIST (https://chemdata.nist.gov/download/peptide_library/libraries/nistmab/NISTmAb_v20190711_mssearch.zip).

Table S3: Overview of identified heavy chain peptides of digested NISTmAb by trypsin-immobilized corundum particles.

Start	Stop	Peptide sequence with modification	m/z	Intensity [a.u.]
1	5	QVTLR/1_1(0,Q,Gln->pyro-Glu)_26eV	599.3524	807,263
41	45	QPPGK/1_1(0,Q,Gln->pyro-Glu)_16eV	509.2705	55,806
41	59	QPPGKALEWLADIWWDDKK/3_1(0,Q,Gln->pyro-Glu)_44eV	760.3849	3,878
46	58	ALEWLADIWWDDK/2_0_42eV	830.9010	7,477
46	59	ALEWLADIWWDDKK/3_0_16eV	596.9702	444,846
46	66	ALEWLADIWWDDKKHYNPSLK/3_0_44eV	876.7778	6,979
60	66	HYNPSLK/1_0_34eV	858.4455	1,577
60	68	HYNPSLKDR/3_1(6,K,Hex)_25eV	431.2234	50,595
67	73	DRLTISK/3_0_11eV	278.1664	21,386
69	73	LTISK/1_0_22eV	561.3627	41,330
69	77	LTISKDTSK/2_0_18eV	496.7857	43,208
74	83	DTSKNQVVLK/2_0_20eV	566.322	58,238
78	83	NQVVLK/1_1(0,N,Deamidated)_28eV	701.4120	1,410
78	99	NQVVLKVTNMDPADTATYYCAR/3_1(19,C,Carbamidomethyl)_36eV	844.0742	16,593
84	99	VTNMDPADTATYYCAR/2_1(13,C,Carbamidomethyl)_33eV	924.8931	99,878
125	136	GPSVFPLAPSSK/1_0_57eV	1186.6392	11,578
222	251	SCDKTHTCPPCPAPELLGGPSVFLFPPKPK/3_3(1,C,Carbamidomethyl) (7,C,Carbamidomethyl)(10,C,Carbamidomethyl)_68eV	1112.2225	9,635
226	251	THTCPPCPAPELLGGPSVFLFPPKPK/3_2(3,C,Carbamidomethyl) (6,C,Carbamidomethyl)_45eV	948.8275	8,475
252	258	DTLMISR/1_0_47eV	835.4325	28,245
259	277	TPEVTCVVVDVSHEDPEVK/2_1(5,C,Carbamidomethyl)_46eV	1070.0233	34,582
278	291	FNWYVDGVEVHNAK/3_1(11,N,Deamidated)_11eV	560.2674	28,134
292	295	TKPR/1_0_24eV	501.3150	5,387
305	320	VVSVLTVLHQDWLNGK/2_0_35eV	904.5095	17,066
305	323	VVSVLTVLHQDWLNGKEYK/3_1(9,Q,Deamidated)_25eV	743.7330	71,880
321	323	EYK/1_0_18eV	439.2167	39,869
324	329	CKVSNK/1_1(0,C,Carbamidomethyl)_29eV	735.3826	11,597
326	337	VSNKALPAPIEK/2_0_23eV	633.8692	40,010
330	337	ALPAPIEK/1_0_34eV	838.5076	36,703
330	341	ALPAPIEKTISK/2_0_23eV	634.3846	20,103
338	341	TISK/1_0_14eV	448.2757	81,588

348	363	EPQVYTLPPSREEMTK/3_0_11eV	635.6582	66,705
359	373	EEMTKNQVSLTCLVK/3_1(11,C,Carbamidomethyl)_13eV	593.9649	37,879
364	373	NQVSLTCLVK/2_2(0,N,Deamidated)(6,C,Carbamidomethyl)_13eV	581.8111	114,604
374	395	GFYPSDIAVEWESNGQPENNYK/3_0_43eV	848.7172	277,848
396	412	TTPPVLDSDGSFFLYSK/2_0_42eV	937.4719	476,495
396	417	TTPPVLDSDGSFFLYSKLTVDK/3_0_33eV	810.7530	55,206
413	417	LTVDK/1_0_25eV	575.3394	61,506
413	419	LTVDKSR/2_0_15eV	409.7398	183,283
420	442	WQQGNVFSCSVMHEALHNHYTQK/3_2(8,C,Carbamidomethyl)(11,M,Oxidation)_51eV	939.7567	2,159
443	449	SLSLSPG/1_0_24eV	660.3574	562,845
214	221	VDKRVEPK/2_0_11eV	485.7870	13,569
324	337	CKVSNKALPAPIEK/3_1(0,C,Pyro-carbamidomethyl)_14eV	513.2830	241,480
326	341	VSNKALPAPIEKTISK/3_0_12eV	566.0042	30,070
330	343	ALPAPIEKTISKAK/2_0_21eV	733.9464	2,771

Table S4: Overview of identified light chain peptides of digested NISTmAb by trypsin-immobilized corundum particles.

Start	Stop	Peptide sequence with modification	m/z	Intensity [a.u.]
1	18	DIQMTQSPSTLSASVGDR/3_0_17eV	631.6417	193,601
1	28	DIQMTQSPSTLSASVGDRVTITCSASSR/3_1(22,C,Carbamidomethyl)_34eV	985.8114	236,177
29	41	VGYMHWYQQKPGK/2_0_47eV	811.3974	104,20
29	44	VGYMHWYQQKPGKAPK/2_0_41eV	959.4990	8,265
45	52	LLIYDTSK/1_0_38eV	952.5327	44,450
45	60	LLIYDTSKLASGVPSR/2_0_37eV	860.4889	48,742
53	60	LASGVPSR/1_0_38eV	786.4428	31,391
103	106	VEIK/1_0_14eV	488.3075	37,214
103	107	VEIKR/1_0_26eV	644.4061	10,452
107	125	RTVAAPSVFIFPPSDEQLK/3_0_24eV	701.3814	97,578
108	125	TVAAPSVFIFPPSDEQLK/2_0_49eV	973.5186	181,525
126	141	SGTASVVCLLNNFYPR/3_2(7,C,Carbamidomethyl)(10,N,Deamidated)_16eV	600.3007	145,189
126	144	SGTASVVCLLNNFYPREAK/2_1(7,C,Carbamidomethyl)_46eV	1063.5380	5,994
142	148	EAKVQWK/2_0_10eV	444.7509	12,182
145	148	VQWK/1_0_16eV	560.3184	38,180
149	168	VDNALQSGNSQESVTEQDSK/3_0_34eV	712.6677	165,683
169	182	DSTYSLSSTLTLSK/2_0_34eV	751.8855	217,659
183	187	ADYEK/1_0_27eV	625.2828	22,503
188	189	HK/1_0_14eV	284.1473	7,738
190	206	VYACEVTHQGLSSPVTK/2_1(3,C,Carbamidomethyl)_41eV	938.4623	19,894

207	210	SFNR/1_0_29eV	523.2613	16,059
207	213	SFNRGEC/1_1(6,C,Carbamidomethyl)_42eV	869.3555	16,631

Table S5: Overview of identified heavy chain peptides of digested NISTmAb with trypsin in solution.

Start	Stop	Peptide sequence with modification	m/z	Intensity [a.u.]
1	5	QVTLR/1_1(0,Q,Gln->pyro-Glu)_26eV	599.3525	393,531
41	45	QPPGK/1_1(0,Q,Gln->pyro-Glu)_16eV	509.2717	2,284
46	59	ALEWLADIWWDDKK/3_0_20eV	596.9683	63,243
84	99	VTNMDPADTATYYCAR/3_2(5,P,Oxidation)(13,C,Carbamidomethyl)_34eV	622.2685	1,530
100	124	DMIFNFYFDVWGQGTTVTVSSASTK/3_0_51eV	934.4382	4,805
222	251	SCDKTHTCPPCPAPELLGGPSVFLFPPKPK/3_3(1,C,Carbamidomethyl)(7,C,Carbamidomethyl)(10,C,Carbamidomethyl)_80eV	1112.2299	1,282
252	258	DTLMISR/1_0_47eV	835.4304	13,988
259	277	TPEVTCVVVDVSHEDPEVK/2_1(5,C,Carbamidomethyl)_46eV	1070.0080	10,826
278	291	FNWYVDGVEVHNAK/2_0_48eV	839.4075	31,382
305	320	VVSVLTVLHQDWLNGK/3_1(9,Q,Deamidated)_29eV	603.6690	89,575
305	323	VVSVLTVLHQDWLNGKEYK/3_1(13,N,Deamidated)_37eV	743.7358	24,917
324	329	CKVSNK/1_1(0,C,Carbamidomethyl)_29eV	735.3775	12,665
326	337	VSNKALPAPIEK/2_0_23eV	633.8711	20,371
330	337	ALPAPIEK/1_0_34eV	838.4962	24,467
338	341	TISK/1_0_14eV	448.2752	83,684
364	373	NQVSLTCLVK/2_2(0,N,Deamidated)(6,C,Carbamidomethyl)_17eV	581.8088	37,528
374	395	GFYPSDIAVEWESNGQPENNYK/3_0_43eV	848.7175	147,547
396	412	TTPPVLDSDGSFFLYSK/2_0_42eV	937.4628	71,712
396	417	TTPPVLDSDGSFFLYSKLTVDK/3_0_22eV	810.74939	13,886
413	417	LTVDK/1_0_25eV	575.3391	30,236
413	419	LTVDKSR/2_0_15eV	409.7399	76,635
420	442	WQQGNVFSCSVMHEALHNHYTQK/3_1(8,C,Carbamidomethyl)_47eV	934.4260	9,698
443	449	SLSLSPG/1_0_24eV	660.3576	468,945
324	337	CKVSNKALPAPIEK/3_1(0,C,Pyro-carbamidomethyl)_14eV	513.2866	73,894

Table S6: Overview of identified heavy chain peptides of digested NISTmAb with trypsin in solution.

Start	Stop	Peptide sequence with modification	m/z	Intensity [a.u.]
1	18	DIQMTQSPSTLSASVGDR/2_0_34eV	946.9506	1,2670
1	28	DIQMTQSPSTLSASVGDRVTITCSASSR/3_1(22,C,Carbamidomethyl)_34eV	985.8145	208,505
29	44	VGYMHWYQQKPGKAPK/2_0_28eV	959.5006	5,555

45	52	LLIYDTSK/1_0_38eV	952.5333	21,000
45	60	LLIYDTSKLASGVPSR/2_0_37eV	860.4840	8,192
53	60	LASGVPSR/1_0_38eV	786.4470	4,906
103	106	VEIK/1_0_14eV	488.3065	2,532
103	107	VEIKR/2_0_10eV	322.7071	107,830
108	125	TVAAPSVFIFPPSDEQLK/3_0_22eV	649.3468	123,079
126	141	SGTASVVCLLNNFYPR/3_2(7,C,Carbamidomethyl)(10,N,Deamidated)_20eV	600.2959	42,210
126	144	SGTASVVCLLNNFYPREAK/3_1(7,C,Carbamidomethyl)_15eV	709.3619	66,338
142	148	EAKVQWK/2_0_10eV	444.7476	21,188
145	148	VQWK/1_0_22eV	560.3131	20,302
145	168	VQWKVDNALQSGNSQESVTEQDSK/3_0_49eV	893.0958	25,089
149	168	VDNALQSGNSQESVTEQDSK/3_0_29eV	712.6613	111,898
169	182	DSTYSLSSTLTLSK/2_0_34eV	751.8812	48,892
183	187	ADYEK/1_0_27eV	625.2803	9,163
188	189	HK/1_0_14eV	284.1615	2,772
207	213	SFNRGEC/1_1(6,C,Carbamidomethyl)_42eV	869.3596	9,932

Reusability of trypsin particles for LC-MS/MS-based Quantification of NISTmAb

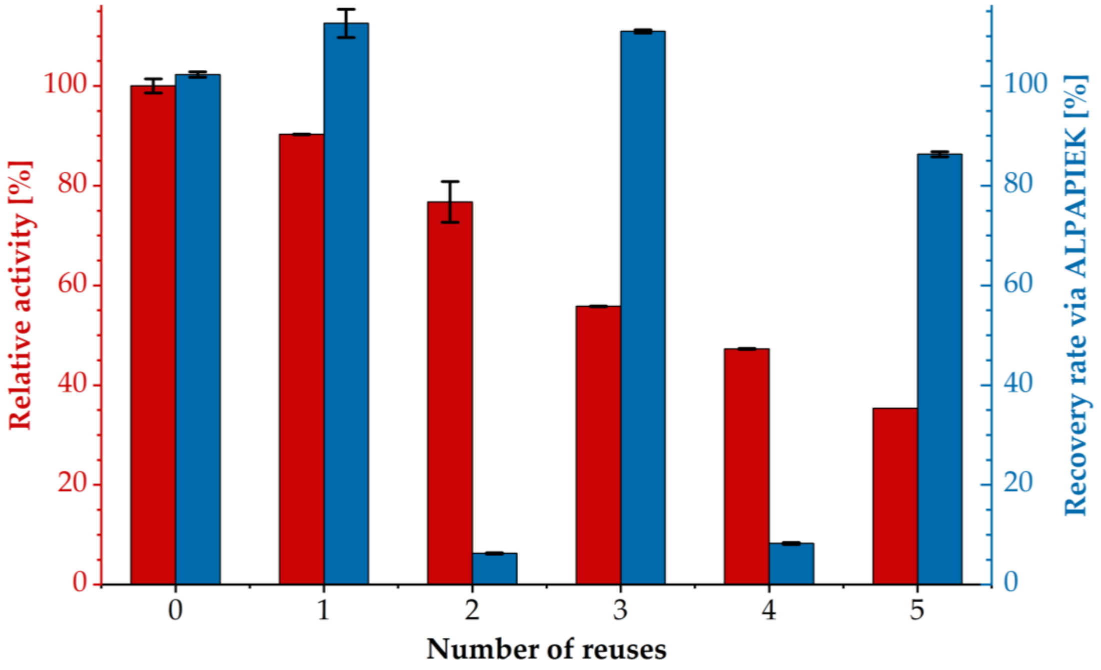

Figure S7: Reusability of immobilized trypsin particles over consecutive digests at 37 °C, monitored recovery rate by LC–MS/MS (blue) and residual enzyme activity in the BAPNA assay (red) including blank runs without antibody (number 2 and 4). Errors were calculated as the standard deviation of duplicate measurements.

ABID 2.0 Evaluation of digested Herceptin with functionalized corundum particles at 60 °C

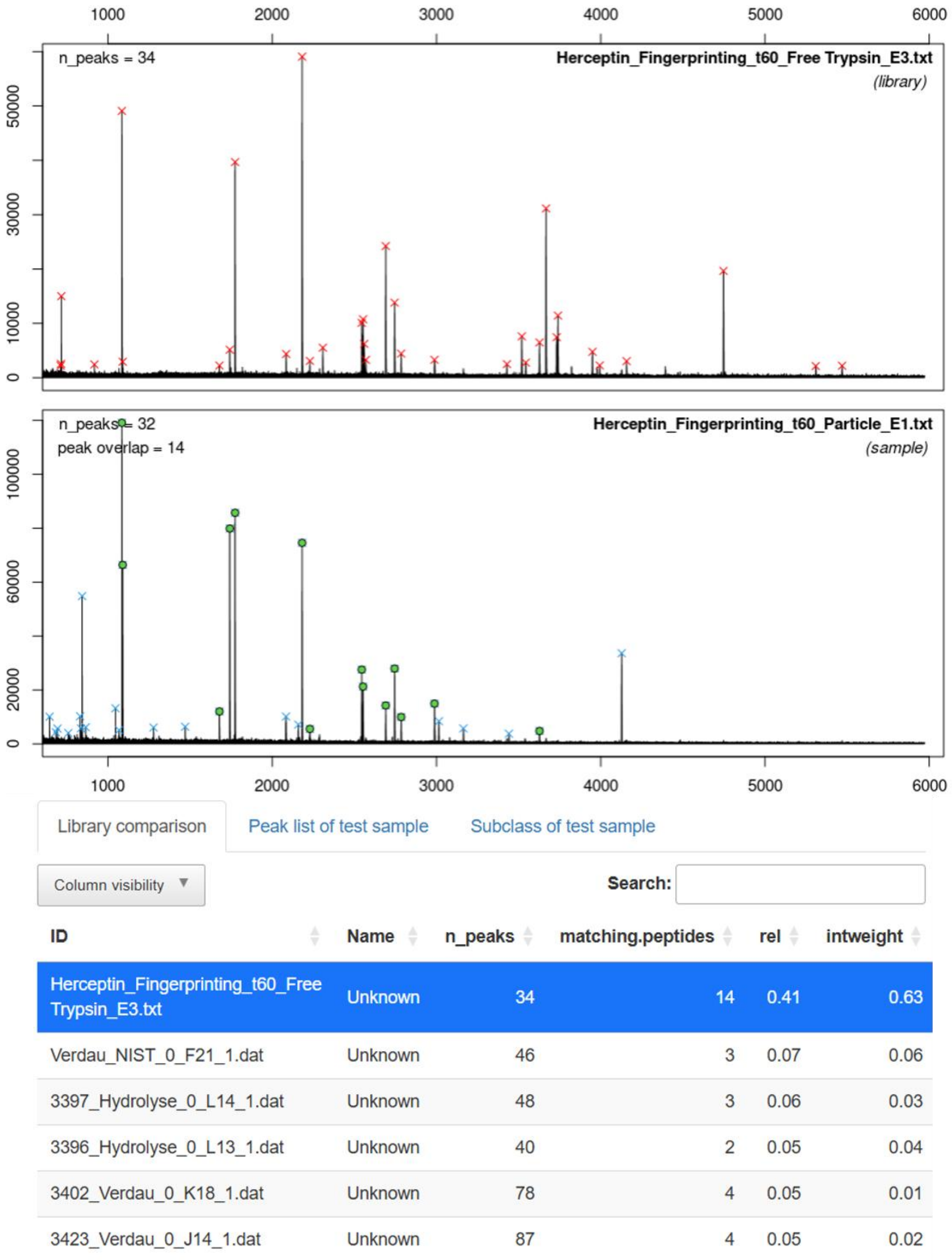

ID	Name	n_peaks	matching.peptides	rel	intweight
Herceptin_Fingerprinting_t60_Free Trypsin_E3.txt	Unknown	34	14	0.41	0.63
Verdau_NIST_0_F21_1.dat	Unknown	46	3	0.07	0.06
3397_Hydrolyse_0_L14_1.dat	Unknown	48	3	0.06	0.03
3396_Hydrolyse_0_L13_1.dat	Unknown	40	2	0.05	0.04
3402_Verdau_0_K18_1.dat	Unknown	78	4	0.05	0.01
3423_Verdau_0_J14_1.dat	Unknown	87	4	0.05	0.02

Figure S8: Comparison of MALDI-TOF MS spectra from digested Herceptin at 60 °C with trypsin-immobilized corundum particles (sample) and trypsin in solution (library) with ABID 2.0 software [64]. The software finds 14 matching peptides. The next best matches only have ≤ 4 matching peptides.

Sequence coverage of peptide mass fingerprinting by tryptic digestion of Herceptin with corundum-functionalized particles

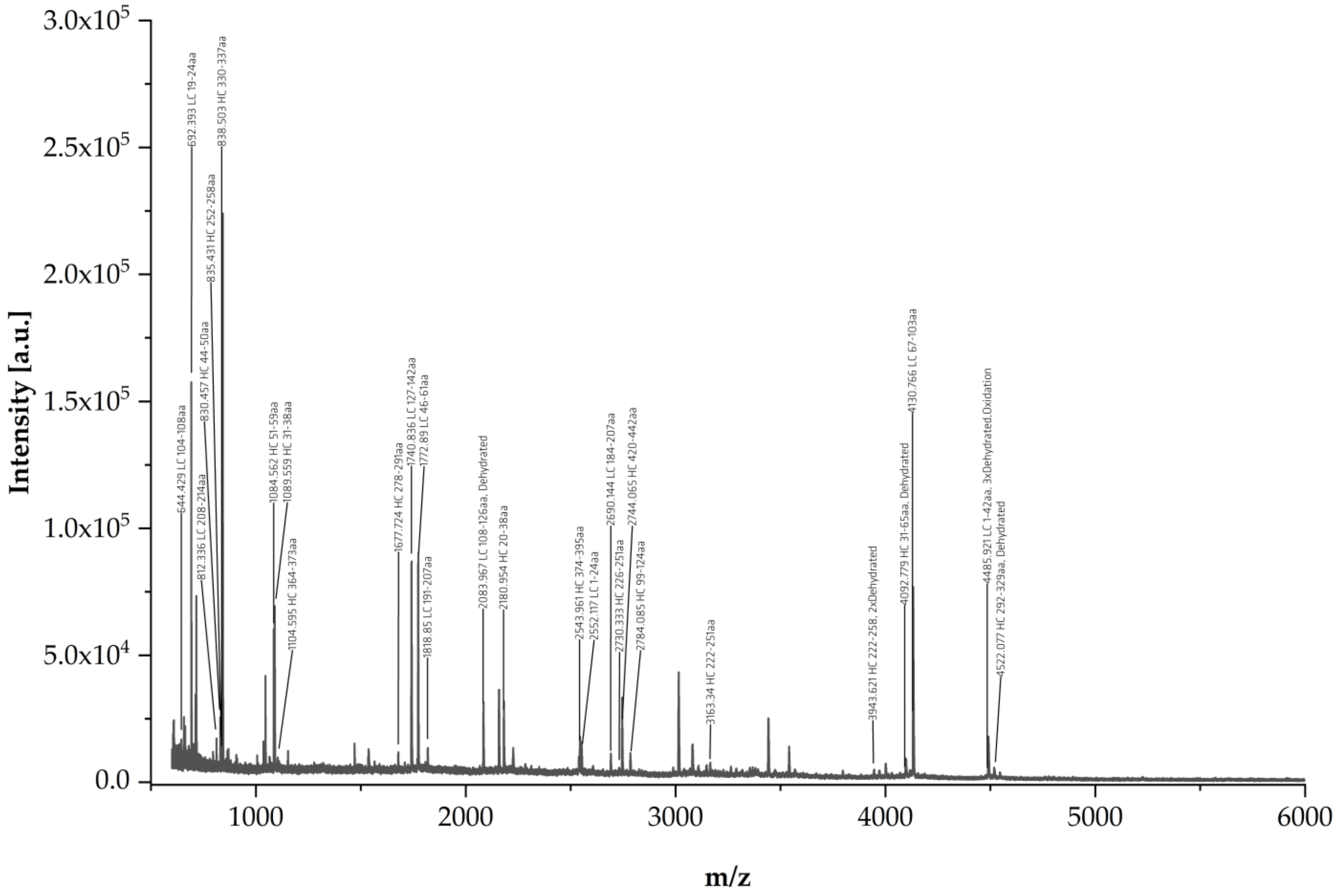

Figure S9: MALDI-TOF fingerprint spectrum of Herceptin after 15 minutes of incubation with TCEP at 99 °C, followed by 60 minutes of incubation with trypsin-functionalized particles at 37 °C. Peptide peaks are assigned to the Herceptin amino acid sequence. The sequence coverage is around 60.3%.
